# CircYthdc2 generates polypeptides through two translation strategies to facilitate virus escape

**DOI:** 10.1007/s00018-024-05148-9

**Published:** 2024-02-15

**Authors:** Weiwei Zheng, Linchao Wang, Shang Geng, Tianjun Xu

**Affiliations:** 1 Laboratory of Fish Molecular Immunology, College of Fisheries and Life Science, Shanghai Ocean University, Shanghai, China; 2 Laboratory for Marine Biology and Biotechnology, Qingdao Marine Science and Technology Center, Qingdao, China; 3 Marine Biomedical Science and Technology Innovation Platform of Lin-Gang Special Area, Shanghai, China

**Keywords:** CircRNA, Coding ability, STING, Ubiquitination, Antiviral immunity

## Abstract

It is known that about 10 circular RNAs (circRNAs) can encode functional polypeptides in higher mammals. However, it is not clear whether the functional polypeptides that can be translated by circRNAs are only the products of the evolution of higher animals, or also widely exist in other lower organisms. In addition, it is also unclear whether the two ways of translating polypeptides using IRES and m^6^A in the one circRNA are exclusive or coexistent. Here, we discovered a novel circRNA derived from the 3′-5′ RNA helicase Ythdc2 (Ythdc2) gene in lower vertebrate fish, namely circYthdc2, which can translate into a 170 amino acid polypeptide (Ythdc2-170aa) through IRES sequence or m^6^A modification, and is involved in antiviral immune of fish. Moreover, SCRV infection can promote circYthdc2 translate Ythdc2-170aa. Then, we found that both Ythdc2-170aa and Ythdc2 can promote the degradation of STING by promoting the ubiquitination modification of K11 and K48 link of STING, and weaken the host’s antiviral innate immunity. Notably, when circYthdc2 is abundant, Ythdc2 preferentially degrades circYthdc2 and no longer promotes the degradation of STING. Further studies have shown that circYthdc2 is highly conserved from lower vertebrates to higher mammals, and human circYthdc2 can also encode the same polypeptide and play a similar function to that of fish circYthdc2. This discovery confirms for the first time that the ability of circRNA to encode functional proteins is evolutionarily conserved, and finds that the ways of polypeptide translation by the same circRNA were diverse, which is of great significance for further elucidating the function and evolution of circRNAs in vertebrates.

**Supplementary Information:**

The online version contains supplementary material available at 10.1007/s00018-024-05148-9.

## Introduction

Circular RNA (circRNA) is a kind of RNA widely existing in the transcriptome of eukaryotes, and different from linear RNA, circRNA is a covalently closed circular RNA generated in the form of back-splicing, which has no 5′ to 3′ polarity [1, 2]. CircRNAs were not first discovered in eukaryotes, but in hepatitis D virus in the 1970s [3]. Subsequently, with the rapid development of high-throughput sequencing, a large number of such back-splicing circRNAs were identified in the transcriptome of eukaryotes [4]. At first, circRNAs were considered as transcriptional noise or transcriptional by-products without much attention [5, 6]. However, in-depth studies have found that circRNA is a key regulatory factor involved in all aspects of eukaryotic life activities, including proliferation, invasion, and metastasis [5, 7, 8]. Of course, circRNAs belong to a class of so-called “noncoding RNA (ncRNA)”, and the definition of ncRNA refers to the collection of RNAs that do not have translation potential, including long-non-coding RNAs (lncRNAs) and microRNAs (miRNAs) [9]. These two kinds of RNAs have been proved to be very important regulatory factors to participate in various physiological and biochemical processes of eukaryotes through a large number of studies. Although the research of ncRNA has been relatively perfect and systematic after decades of research, our understanding of the function and mechanism of ncRNA regulating life activities may only be the tip of the iceberg, and new functions and mechanisms have been discovered constantly. For example, some lncRNAs and pri-miRNAs found in recent years can encode polypeptides, which is undoubtedly a surprising discovery [10, 11]. In recent years, circRNAs have also been found to encode functional polypeptides and play a certain function. Such as the spanning junction open reading frame in circ-FBXW7 driven by internal ribosome entry site encodes a novel 21-kDa protein, which reduces the half-life of c-Myc by antagonizing USP28-induced c-Myc stabilization in mammals [12]. Moreover, circRNA Circ-EIF6 encodes EIF6-224aa to promote triple-negative breast cancer progression via stabilizing MYH9 and activating Wnt/beta-catenin pathway in mammals [13]. In addition, a novel tumor suppressor protein encoded by circular AKT3 RNA inhibits glioblastoma tumorigenicity by competing with active phosphoinositide-dependent Kinase-1 in mammals [14].

The research into circRNA translation mentioned above only refers to the research carried out in higher mammals, such as mice or humans. However, it is still unknown whether there is circRNA that can translate polypeptides in invertebrates and lower vertebrates, and that is essential for the systematic understanding of the role and generation mechanism of circRNAs in eukaryotes. Moreover, current research shows that there are two main forms of how circRNA translates polypeptides. One is to promote the combination of initiation factor or ribosome with circRNA through the Internal Ribosome Entry Sites (IRES) element sequence, which leads to the beginning of translation [15–21]. Another form is through N6-methyladenosine (m^6^A) modification of circRNA, and m6a-modified circRNA can effectively start translation [22, 23]. Under normal circumstances, the translation of circRNAs is limited and inefficient, but the current study shows that circRNAs can initiate the IRES-mediated efficient translation under heat shock [24, 25] or starvation [26]. However, it is still unclear under what conditions the translation mediated by m^6^A modification is conducted. In addition, it is also unclear whether a circRNA has several different translation modes at the same time, such as IRES sequence-mediated translation or m^6^A-mediated translation. If IRES sequence and m^6^A modification can co-mediate the translation of the same circRNA, it is worth exploring under what conditions which translation mode is initiated. In addition to the above, whether there are other ways to mediate circRNA translation is still unclear, because many circRNAs that can bind to ribosomes do not contain IRES sites and m^6^A sites. Therefore, we want to know whether the translation of circRNA can be activated by some external stress, such as viral infection. These problems mentioned above are all worth exploring urgently. In addition to the translation mode of circRNAs, the functions of the translation products of circRNAs are also poorly understood, as only more than 10 circRNAs capable of translating polypeptides have been identified so far [27].

In response to the above problems, we conducted related explorations in this study, and selected representative lower vertebrate fish as research models to confirm whether circRNA translation polypeptides are evolutionary products of higher animals or are widely present in all vertebrates. By RNA Sequencing (RNA-Seq) and Ribosome Sequencing (Ribo-Seq), we first discovered a novel circRNA, called circYthdc2, which could encode a polypeptide involved in antiviral responses in teleost fish, miiuy croaker (*Miichthys miiuy*). Through further study, we found that circYthdc2 contains an open reading frame (ORF) driven by the internal ribosome entry site (IRES) and m^6^A site, and encodes a functional polypeptide of 170 amino acids which named this polypeptide Ythdc2-170aa. Further studies show that METTL3 and METTL14 act as “writers” to perform m^6^A modification on circYthdc2, while FTO acts as “eraser” to demethylate circYthdc2, and YTHDF1 and YTHDF3 act as “readers” to jointly promote the translation of polypeptides by circYthdc2. We found that Ythdc2-170aa can further inhibit STING-mediated antiviral immune response by targeting STING, thereby promoting RNA viral replication. We found that Ythdc2 protein has the same function as Ythdc2-170aa, and they can promote the ubiquitination modification of K11 and K48 link of STING, promote STING degradation, and weaken the host antiviral immune. Since Ythdc2 is a “read” protein involved in m^6^A modification, we further explored whether Ythdc2 is involved in the translation process of circYthdc2. The results showed that Ythdc2 does not participate in the translation process of circYthdc2, but it can inhibit its translation into polypeptides by degrading circYthdc2. When circYthdc2 is abundant, Ythdc2 preferentially degrades circYthdc2 and no longer promotes the degradation of STING. In addition, we found that circYthdc2 also exists in other vertebrates through prediction and can translate polypeptides with highly conserved amino acid sequences, and verified the existence of human circYthdc2 translation products through experiments. This discovery confirms for the first time that the ability of circRNA to encode functional proteins is evolutionarily conserved, and finds that the ways of polypeptides translation by the same circRNA were diverse, which is of great significance for further elucidating the function and evolution of circRNAs in vertebrates.

## Results

### Characterization of circYthdc2 involved in antiviral immunity

**Fig. 1:**
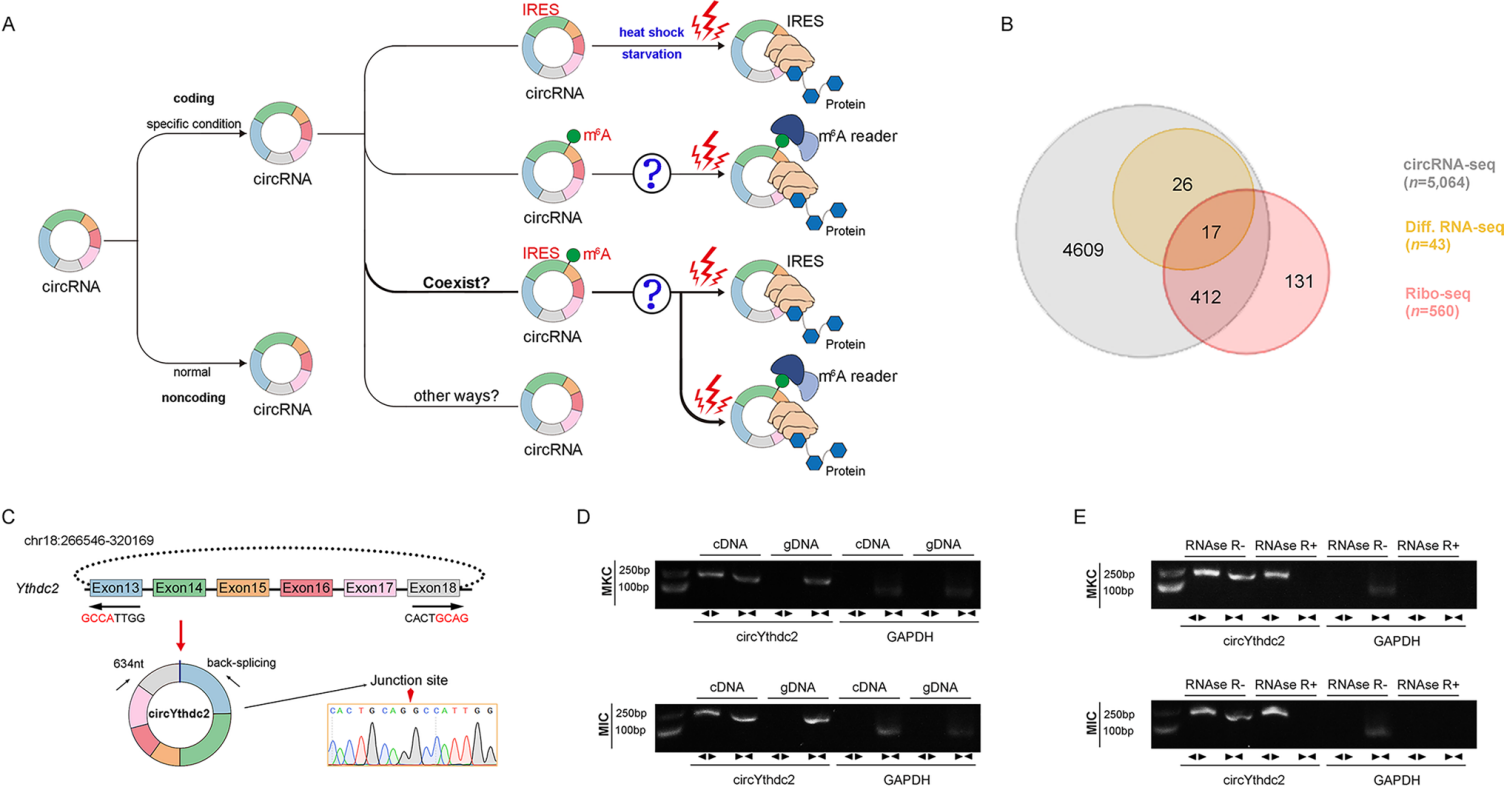
Expression profiles and characterization of circYthdc2. **A** Schematic diagram of circRNA translation ways and its selection conditions. **B** Strategies used for circRNA-seq and ribosome profiling (Ribo-seq). The gray strips represented the total circRNAs by circRNA-Seq. The orange strips represented the differential circRNAs upon SCRV treatment. The red strips represented the circRNAs with potential translation ability by Ribo-seq. **C** We confirmed the head-to-tail splicing of circYthdc2 in the circYthdc2 RT-PCR product by Sanger sequencing. **D** RT-PCR validated the existence of circYthdc2 in MKC and MIC cell lines. CircYthdc2 was amplified by divergent primers in cDNA but not gDNA. GAPDH was used as a negative control. **E** The expression of circYthdc2 and linear Ythdc2 mRNA in both MKC and MIC cell lines was detected by RT-PCR assay followed by nucleic acid electrophoresis or qPCR assay in the presence or absence of RNase R. All data represented the three independent triplicated experiments

The above-mentioned question about whether circRNA translation is widespread, its translation mode and activation ways are still unknown to a large extent (Fig. 1A), and we are very interested in these currently unknown questions, so we have studied the above-mentioned problems by using the representative fish of lower vertebrates as a model. First, we treated miiuy croaker with SCRV virus, then performed RNA-seq analysis to compare the circRNA expression levels between SCRV-treated and untreated spleen samples (GenBank accession no. PRJNA685924). In total, 5,064 distinct circRNA candidates were found. At the same time, we also carried out Ribo-Seq and obtained 560 candidate circRNAs. By crossing with the differential groups obtained from the above SCRV infection, we obtained some candidate circRNAs (Fig. 1B). Among them, we found circular Ythdc2 (circYthdc2) RNA was most differentially expressed and had coding potential.

The transcriptome sequencing revealed that circYthdc2 was 634nt in length (Fig. 1C). Using miiuy croaker whole genome library to perform blast analysis on the host gene Ythdc2 [28, 29], it was found that the Ythdc2 gene was located on chromosome 18, which was composed of 30 exons, and circYthdc2 was self-cyclization from exon13 to exon18. To confirm the objective existence of circYthdc2, first, circYthdc2 divergent primers were designed for RT-PCR amplification, and the amplified products were subjected to Sanger sequencing to confirm that circYthdc2 was spliced from the head to the tail (Fig. 1C). Then, we used convergent primers to amplify Ythdc2 gene and divergent primers to amplify circYthdc2. cDNA and gDNA were extracted separately from MKC and *M. miiuy* intestine cells (MIC) and subjected to RT-PCR and agarose gel electrophoresis assays. The results shown in Fig. 1D indicated that circYthdc2 was amplified from cDNA using only divergent primers, whereas no amplification product was observed from gDNA. Considering that stability was a crucial characteristic of circRNAs, we thus employed RNase R to confirm the stability of circYthdc2. The results from the agarose gel electrophoresis assay showed that circYthdc2, rather than linear Ythdc2 or GAPDH, resisted digestion by RNase R (Fig. 1E). Among the aforementioned cell lines, MKC and MIC cells showed the lowest and highest expression of circYthdc2, respectively. To confirm the reliability of this result, we conducted in vivo and in vitro experiments to detect the changes in the expression level of circYthdc2 under SCRV infection. At the same time, we also detected the expression of the host gene of this circRNA, Ythdc2, after SCRV infection. The results of qPCR experiments showed that Ythdc2 was significantly up-regulated in miiuy croaker spleen tissue treated with SCRV and Poly (I: C) at different time points (Fig. S1A). The level of circYthdc2 significantly increases during the initial period of SCRV infection or Poly (I: C) stimulation, but subsequently undergoes a significant decrease (Fig. S1A). In addition, SCRV-treated *M. miiuy* kidney cells (MKC) further confirmed the significant expression of circYthdc2 and Ythdc2 (Fig. S1B). We then evaluated the expression levels of circYthdc2 in MKC, *M. miiuy* spinal marrow cells (MSpC), MIC, *M. miiuy* brain cells (MBrC), *M. miiuy* muscle cells (MMC), and *M. miiuy* liver cells (MLC), and these cell line samples were divided into two groups, one treated with RNase R and the other untreated. The mRNA expression of Ythdc2 decreased significantly after RNase R treatment in all aforementioned cell lines, while the expression of circYthdc2 almost did not decrease in all aforementioned cell lines (Fig. S1C). Therefore, we selected MKC and MIC to investigate the function and regulatory mechanism of circYthdc2. Moreover, the half-time of the circYthdc2 transcript was significantly longer than Ythdc2 mRNA after being treated with actinomycin D, which suppressed RNA transcription (Fig. S1D).

In addition, we detected the distribution of circYthdc2 by cytoplasmic nuclear fractionation experiments and found that circYthdc2 was primarily localized in the cytoplasm (Supplementary Fig. 1E). Accordingly, these results suggested that circYthdc2 was a stable circRNA expressed and primarily distributed in the cytoplasm.

### circYthdc2 encodes a 170 amino acid (aa) novel protein, Ythdc2-170aa

**Fig. 2:**
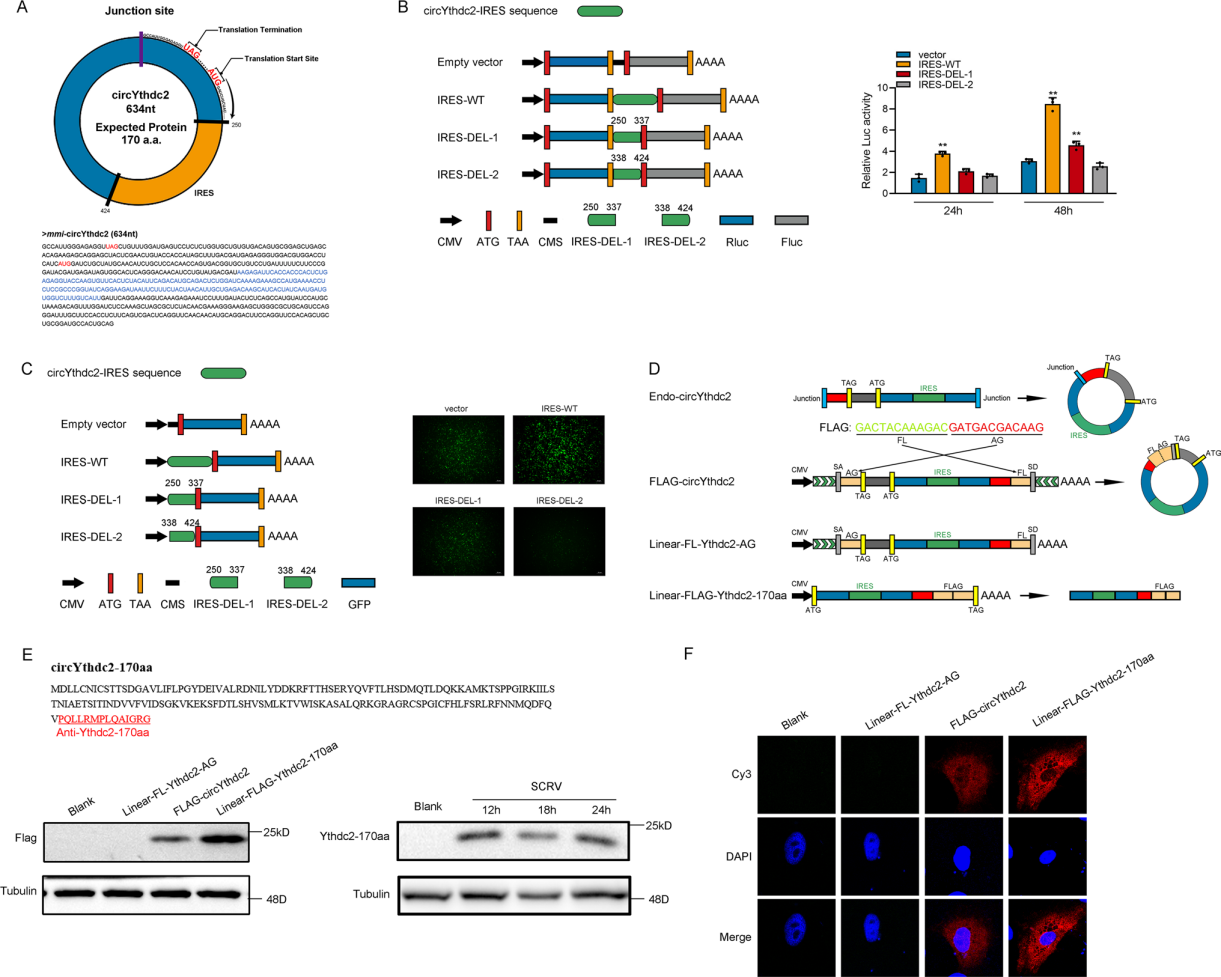
CircYthdc2 encodes a 170 amino acid (aa) novel protein, Ythdc2-170aa. **A** Upper panel, the putative ORF in circYthdc2. Lower panel, the sequences of the putative ORF are shown. **B** The putative IRES activity in circYthdc2 was tested. **C** Left panel: Full-length or truncated circYthdc2 IRES sequences were cloned before GFP as indicated to construct reporter plasmids. Right panel: The empty vector, and full-length or truncated IRES vector were cotransfected with si-eif4E into HEK293 cells, and GFP signals were detected. **D** Schematic diagram of FLAG-circYthdc2, Linear-FL-Ythdc2-AG, and Linear-FLAG-Ythdc2-170aa plasmid construction. **E** Upper panel: The putative Ythdc2-170aa amino acid sequences and antibody generation region were shown as indicated to produce the Ythdc2-170aa antibody. The red amino acids were distinctly formed by the circYthdc2 junction. Lower: FLAG tag antibody was used to detect Ythdc2-170aa expression in MKC cells transfected with the vectors mentioned in Fig. 2D. In addition, Ythdc2-170aa antibody was used to detect Ythdc2-170aa expression in MKC cells after SCRV infection **F** FLAG-circYthdc2, Linear-FL-Ythdc2-AG, and Linear-FLAG-Ythdc2-170aa plasmids were transfected into MKC cells. Immunofluorescence staining using anti-Flag was performed to show the Ythdc2-170aa cellular localization. Original magnification is 630; all data represent the means ± SE from three independent triplicate experiments. *, *p* < 0.05; **, *p* < 0.01

circYthdc2 has a predicted ORF, which may encode a 170 aa protein using an overlapping start-stop codon “UAG……AUG” (Fig. 2A). Through prediction, we found that IRES sequence exists on circYthdc2. Therefore, to confirm this, we carried out a related dual-luciferase assay and GFP protein activity assay to detect IRES activity, and the experimental results show that the complete IRES sequence predicted from circYthdc2 does have the activity of mediating translation. Once the IRES sequence is deleted, it will lead to a significant decrease in activity (Fig. 2B and C). We constructed the Linear-FL-Ythdc2-AG overexpression plasmid, FLAG-circYthdc2 overexpression plasmid, and a linearized Ythdc2-170aa overexpression plasmid, and the construction pattern of these plasmids is shown in Fig. 2D. To confirm that the 170 aa predicted protein was encoded by circYthdc2, we used the Linear-FL-Ythdc2-AG overexpression plasmid, FLAG-circYthdc2 overexpression plasmid, and a linearized Ythdc2-170aa overexpression plasmid. We used an antibody against the Ythdc2-170aa, which should recognize Ythdc2-170aa. In MKC cells, which have lower endogenous circYthdc2 levels, the transfection circYthdc2 and the linearized Ythdc2-170aa plasmid both resulted in the predicted Ythdc2-170aa band, while the overexpression Linear-FL-Ythdc2-AG plasmid did not. In addition, the Ythdc2-170aa polypeptide cannot be detected under normal conditions, but we found that the Ythdc2-170aa polypeptide was generated in large quantities under the condition of SCRV virus infection (Fig. 2E). These results prove that Ythdc2-170aa encoded this 170aa novel protein, which we termed Ythdc2-170aa. Immunofluorescence using an anti-flag antibody confirmed the cytoplasmic localization of Linear-FLAG-Ythdc2-170aa in MKC cells, as shown in Fig. 2F.

### CircYthdc2 and Ythdc2-170aa inhibit host antiviral innate immunity

**Fig. 3:**
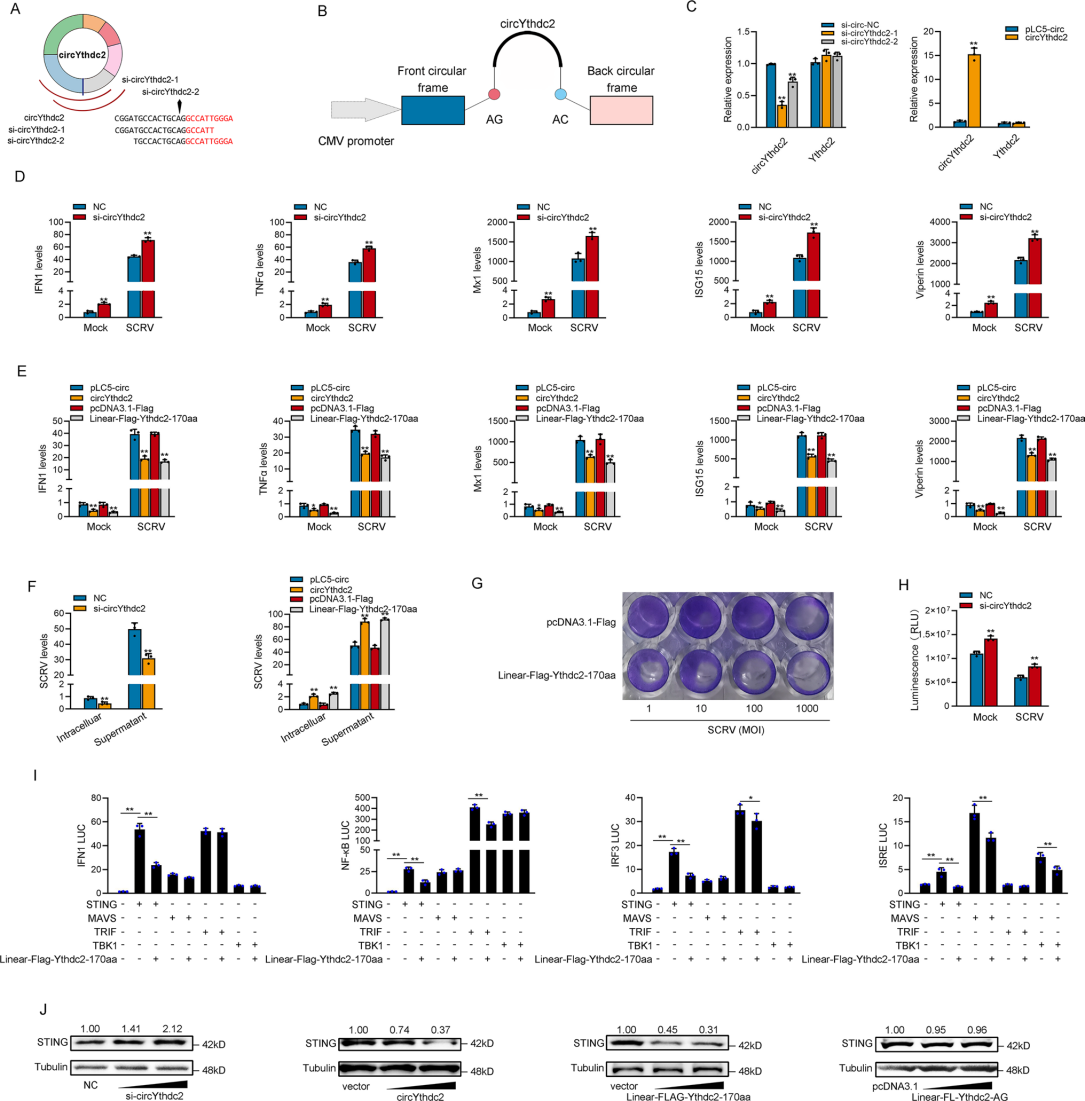
CircYthdc2 and Ythdc2-170aa inhibit host antiviral innate immunity. **A** and **B** The schematic diagram of siRNAs (**A**) and circYthdc2 overexpression plasmid structure (**B**). **C** qPCR analysis of circYthdc2 and linear Ythdc2 mRNA in MIC cells treated with siRNAs. qPCR analysis of circYthdc2 and linear Ythdc2 mRNA in MKC cells overexpressing circYthdc2. **D** and **E** qPCR assays were performed to determine the expression levels of IFN1, TNF-α, Mx1, ISG15, and Viperin in MIC cells transfected with NC or si-circYthdc2 (**D**) and transfected in MKC cells with circYthdc2 or pLC5-circ and Linear-FLAG-Ythdc2-170aa or pcDNA3.1-FLAG after SCRV infection (**E**). **F** circYthdc2 and Ythdc2-170aa promote SCRV replication. MIC cells transfected with NC or si-circYthdc2 and MKC cells were transfected with pLC5-circ or circYthdc2 and pcDNA3.1-FLAG or Linear-FLAG-Ythdc2-170aa for 24 h, respectively, then infected with SCRV at 24 h. The qPCR analysis was conducted for intracellular and supernatant SCRV RNA expression. **G** MKC cells seeded in 48-well plates overnight were treated with cultural supernatants at the dose indicated for 48 h. Then, cell monolayers were fixed with 4% paraformaldehyde and stained with 1% crystal violet. MKC cells were transfected with Linear-FLAG-Ythdc2-170aa or pcDNA3.1-FLAG. **H** Effect of circYthdc2 on cell viability after SCRV infection. MIC cells were transfected with NC or si-circYthdc2 for 24 h and then treated with SCRV for 24 h. Cell viability assay was measured. **I** Ythdc2-170aa counteracts the negative effect of STING. Relative luciferase activities were detected in MKC after cotransfection with STING, MAVS, TRIF, and TBK1 expression plasmid, pRL-TK *Renilla* luciferase plasmid, luciferase reporters, pcDNA3.1-FLAG or Linear-FLAG-Ythdc2-170aa. **J** Relative protein levels of STING in MIC cells after transfected with NC or si-circYthdc2 and in MKC cells with pLC5-circ or circYthdc2 and pcDNA3.1-FLAG or Linear-FLAG-Ythdc2-170aa or Linear-FL-Ythdc2-AG. All data represented the mean ± SE from three independent triplicated experiments. *, *p* < 0.05; **, *p* < 0.01

We designed the two small interfering RNAs (siRNA) against circYthdc2, and the overexpression plasmid of circYthdc2 was constructed to explore the biological function of circYthdc2 (Fig. 3A and B). Consequently, two siRNAs (si-circYthdc2-1 and si-circYthdc2-2) decreased the circYthdc2 expression level, but such siRNAs did not affect the expression level of linear Ythdc2 mRNA in MKC. As si-circYthdc2-1 could induce higher inhibitory efficiency; thus, we selected si-circYthdc2-1 (termed si-circYthdc2) for the subsequent experiment (left panel of Fig. 3C). Moreover, the circYthdc2 overexpression plasmid was successfully constructed, as it significantly increased the circYthdc2 expression levels rather than linear Ythdc2 mRNA in MKC cells (right panel of Fig. 3C). Considering that IFN and ISGs are important antiviral effectors, we focused on investigating the role of circYthdc2 and Ythdc2-170aa in regulating the expression of IFN, ISGs, and inflammatory cytokines. As shown in Fig. 3D, the silence of circYthdc2 increased the expression levels of these genes under SCRV treatment. By contrast, the overexpression of circYthdc2 or Linear-FLAG-Ythdc2-170aa both could significantly inhibit the expression levels of interferon IFN1, inflammatory cytokines (TNF-α), and antiviral genes such as myxovirus resistance protein 1 (Mx1), ISG15, and Viperin after SCRV infection (Fig. 3E).

Furthermore, we examined the effect of circYthdc2 and Ythdc2-170aa on SCRV replication to explore the biological significance of circYthdc2 and Ythdc2-170aa in SCRV-induced host cells. Detecting SCRV RNA level by qPCR, we found that silence of circYthdc2 significantly inhibited SCRV replication after 24 h of SCRV infection (left panel of Fig. 3F). In addition, both circYthdc2 and Linear-FLAG-Ythdc2-170aa overexpression significantly promoted SCRV replication after 24 h of SCRV infection (right panel of Fig. 3F). In addition, SCRV replication was also monitored by the appearance of CPE (cytopathic effect) in MKC cells. As shown in Fig. 3G, overexpression of Linear-FLAG-Ythdc2-170aa presented more CPE in the cells. These results demonstrated that Ythdc2-170aa can promote SCRV replication. When we investigated the effect of circYthdc2 on the cell viability of MIC cells, we found that the silence of circYthdc2 significantly increased cell viability compared with the control group after 24 h of SCRV infection (Fig. 3H). We want to know which gene in the antiviral signal pathway of teleost is affected by Ythdc2-170aa, so we tested STING, MAVS, TRIF, and TBK1 through the double luciferase reporter gene system. Then, the STING, MAVS, TRIF, and TBK1 plasmid with only CDS; Ythdc2-170aa plasmid; and various reporter gene plasmids were cotransfected into EPC cells. The results showed that Ythdc2-170aa inhibited the activity of reporter genes such as IFN1, NF-κB, IRF3, and ISRE commonly by affecting the activity of STING (Fig. 3I). In order to further verify the correctness of the results of double luciferase experiment, we detected the effect of circYthdc2 on STING protein by Western blotting in MKC and MIC cells. The result shows that silence of circYthdc2 significantly increased the protein levels of STING, and overexpression circYthdc2 and Ythdc2-170aa both significantly decreased the protein levels of STING, and overexpression Linear-FL-Ythdc2-AG did not have any effect to STING protein (Fig. 3J). Collectively, circYthdc2 could inhibit STING-mediated antiviral immune response by promoting the degradation of STING protein, and all the experimental results show that the inhibitory effect of circYthdc2 on antiviral immune response mainly comes from the role of its encoded polypeptide Ythdc-170aa.

### Ythdc2 inhibits host antiviral innate immunity

**Fig. 4:**
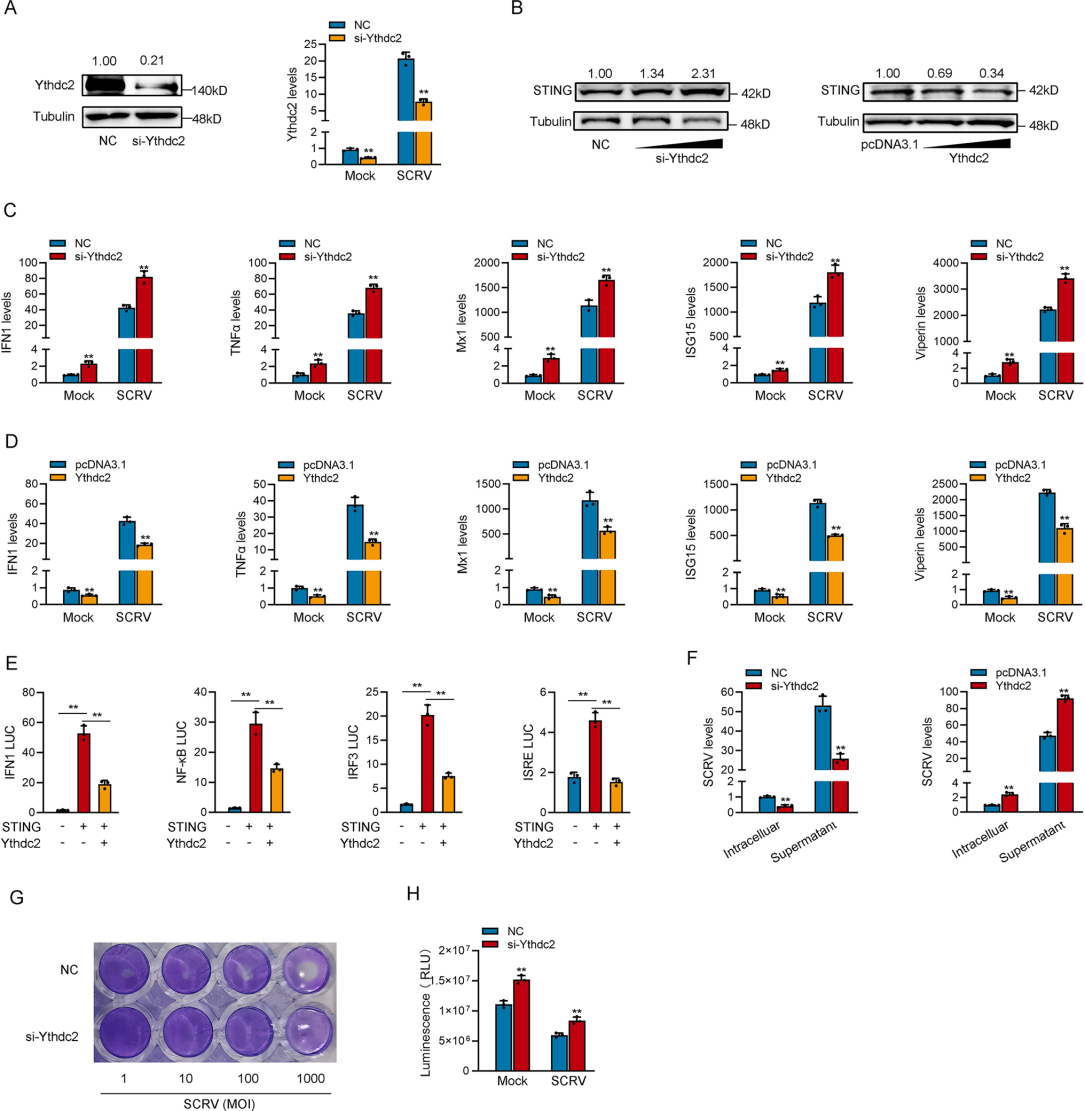
Ythdc2 inhibits host antiviral innate immunity. **A** Relative protein and mRNA levels of Ythdc2 in MIC cells after transfected with NC or si-circYthdc2. **B** Relative protein levels of STING in MIC cells after transfected with NC or si-Ythdc2 and in MKC cells with pcDNA3.1 or Ythdc2. **C** and **D** qPCR assays were performed to determine the expression levels of IFN1, TNF-α, Mx1, ISG15, and Viperin in MIC cells transfected with NC or si-Ythdc2 (**C**) and transfected in MKC cells with pcDNA3.1 and Ythdc2 after SCRV infection (**D**). **E** Ythdc2 counteracts the negative effect of STING. Relative luciferase activities were detected in MKC after cotransfection with STING expression plasmid, pRL-TK *Renilla* luciferase plasmid, luciferase reporters, pcDNA3.1, Ythdc2. **F** Ythdc2 promotes SCRV replication. MIC cells transfected with NC or si-Ythdc2 and MKC cells were transfected with pcDNA3.1 or Ythdc2 for 24 h, respectively, then infected with SCRV at 24 h. The qPCR analysis was conducted for intracellular and supernatant SCRV RNA expression. (G) MIC cells seeded in 48-well plates overnight were treated with cultural supernatants at the dose indicated for 48 h. Then, cell monolayers were fixed with 4% paraformaldehyde and stained with 1% crystal violet. MIC cells were transfected with NC or si-Ythdc2. **H** Effect of Ythdc2 on cell viability after SCRV infection. MIC cells was transfected with NC or si-Ythdc2 for 24 h and then treated with SCRV for 24 h. Cell viability assay were measured. All data represented the mean ± SE from three independent triplicated experiments. *, *p* < 0.05; **, *p* < 0.01

Through prediction, we know that Ythdc2-170aa has only one HELICc domain, and Ythdc2, the host gene of circYthdc2, also has this domain, and their amino acid sequences are consistent. Therefore, we suspect that they may have the same function. We designed the siRNA against Ythdc2, and the overexpression plasmid of Ythdc2 was constructed to explore the biological function of Ythdc2. Consequently, the siRNA (si-Ythdc2) decreased the Ythdc2 protein level and mRNA level in MKC cells (Fig. 4A). We detected whether Ythdc2 could affect the expression level of STING protein by Western blotting. As we predicted, the silence of Ythdc2 significantly increased the protein levels of STING, and overexpression of Ythdc2 significantly decreased the protein levels of STING (Fig. 4B). We also focused on investigating the role of Ythdc2 in regulating the expression of IFN, ISGs, and inflammatory cytokines. As shown in Fig. 4C, the silence of Ythdc2 increased the expression levels of these genes under SCRV treatment. By contrast, the overexpression of Ythdc2 could significantly inhibit the expression levels of interferon IFN1, TNF-α, Mx1, ISG15, and Viperin after SCRV infection (Fig. 4D). Then, the STING plasmid with only CDS, Ythdc2 plasmid, and various reporter gene plasmids were cotransfected into EPC cells. The results show that Ythdc2 inhibited the activity of reporter genes such as IFN1, NF-κB, IRF3, and ISRE commonly by affecting the activity of STING (Fig. 4E).

Furthermore, we examined the effect of Ythdc2 on SCRV replication to explore the biological significance of Ythdc2 in SCRV-induced host cells. Through qPCR detection of SCRV RNA levels, we observed that silencing of Ythdc2 significantly inhibited SCRV replication 24 h after SCRV infection, resulting in a decrease of approximately 2/3 in SCRV levels (left panel of Fig. 4F). In addition, the Ythdc2 overexpression significantly promoted SCRV replication after 24 h of SCRV infection, resulting in an approximately twofold increase in SCRV levels (right panel of Fig. 4F). In addition, SCRV replication was also monitored by the appearance of CPE (cytopathic effect) in MIC cells. As shown in Fig. 4G, Knockdown of the Ythdc2 produced less CPE in the cells. These results demonstrated that Ythdc2 can enhance SCRV replication. When we investigated the effect of Ythdc2 on the cell viability of MIC cells, we found that the silence of Ythdc2 significantly increased cell viability after 24 h of SCRV infection (Fig. 4H). Collectively, Ythdc2 could inhibit STING-mediated antiviral immune response by promoting the degradation of STING protein, and all the results show that Ythdc2 has the same function of inhibiting host antiviral immune response as Ythdc2-170aa.

### Ythdc2-170aa and Ythdc2 both promoted K48 and K11-linked ubiquitination of STING

**Fig. 5:**
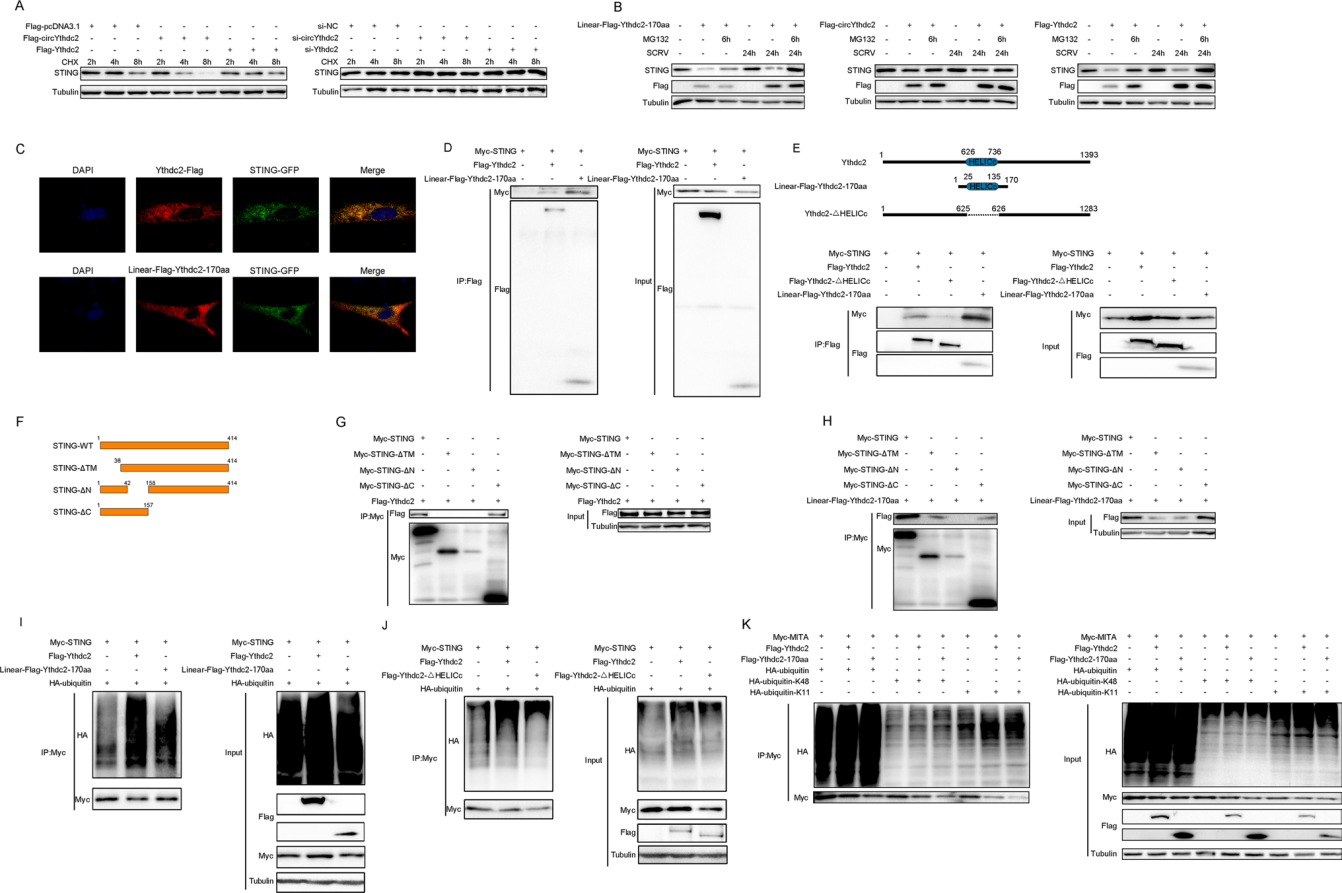
Ythdc2-170aa and Ythdc2 both promoted K11 and K48-linked ubiquitination of STING. **A** MKC cells were transfected with Flag-Ythdc2 and Flag-circYthdc2 plasmids, the cells were treated with 10 μM CHX for a different time before immunoblot analysis was performed; MIC cells were silence Ythdc2 or circYthdc2, and the cells were treated with 10 μM CHX for a different time before immunoblot analysis was performed **B** MKC cells were transfected with Linear-Flag-Ythdc2-170aa or Flag-circYthdc2 or Flag-Ythdc2 plasmids, after 42 h, the cells were treated with DMSO or 10 μM MG132 for 6 h before immunoblot analysis was performed. **C** Flag-Ythdc2 and Linear-Flag-Ythdc2-170aa were cotransfected with GFP-STING into MKC cells. Immunofluorescence staining using anti-Flag was performed to show the Ythdc2 and STING or Ythdc2-170aa and STING cellular localization. Original magnification is 630. **D** Immunoprecipitation and immunoblot analysis of Flag-Ythdc2 or Linear-Flag-Ythdc2-170aa with Myc-STING, in EPC cells. IP, immunoprecipitation. **E** Upper panel: Schematic diagram of Ythdc2-△HELICc plasmid construction. Lower panel: Immunoprecipitation and immunoblot analysis of Flag-Ythdc2, Flag-Ythdc2-△HELICc or Linear-Flag-Ythdc2-170aa with Myc-STING, in MKC cells. IP, immunoprecipitation. **F** Schematic diagram of STING, STING-△TM, STING-△N, STING-△C plasmid construction. **G** Immunoprecipitation and immunoblot analysis of Flag-Ythdc2, with Myc-STING, Myc-STING-△TM, Myc-STING-△N, Myc-STING-△C in MKC cells. IP, immunoprecipitation. **H** Immunoprecipitation and immunoblot analysis of Linear-Flag-Ythdc2-170aa with Myc-STING, Myc-STING-△TM, Myc-STING-△N, Myc-STING-△C in MKC cells. IP, immunoprecipitation. **I** Coimmunoprecipitation analysis of STING ubiquitination in EPC cells transfected with Myc-STING or HA-ubiquitin-WT in the presence of control vector, Flag-Ythdc2 or Linear-Flag-Ythdc2-170aa expression plasmid. IP, immunoprecipitation. **J** Coimmunoprecipitation analysis of STING ubiquitination in EPC cells transfected with Myc-STING or HA-ubiquitin-WT in the presence of control vector, Flag-Ythdc2, or Flag-Ythdc2-△HELICc, or Linear-Flag-Ythdc2-170aa expression plasmid. IP, immunoprecipitation. **K** Coimmunoprecipitation analysis of STING ubiquitination in MKC cotransfected with Myc-STING, Flag-Ythdc2 or Linear-Flag-Ythdc2-170aa expression plasmid and HA-ubiquitin-WT, HA-ubiquitin-K11 or HA-ubiquitin-K48 plasmids. All data represented the three independent triplicated experiments

To determine which way was used to promote STING degradation by Ythdc2-170aa and Ythdc2. Therefore, we first explored the effects of circYthdc2 and Ythdc2 on the STING protein. As shown in Fig. 5A, the results show that overexpression of circYthdc2 or Ythdc2 could promote STING protein degradation, while silence of circYthdc2 or Ythdc2 could inhibit STING protein degradation. To further explore how sting protein is degraded, we tried to use protease inhibitors to block the degradation of circYthdc2 and Ythdc2 on STING. The results are shown in Fig. 5B, the protease inhibitor MG132 can effectively prevent circYthdc2 and Ythdc2 from degrading STING protein. In addition, we found that both circYthdc2 and Linear-FLAG-Ythdc2-170aa could effectively block the degradation of STING by MG132, and this result suggests that Ythdc2-170aa, the translation product of circYthdc2, may play a role in degrading STING. These results indicated that Ythdc2-170aa or Ythdc2 may promote STING degradation through the proteasome pathway. Immunostaining with Flag-specific antibodies shows a very high degree of colocalization both between Linear-FLAG-Ythdc2-170aa and GFP-STING or Flag-Ythdc2 and GFP-STING transfected into MKC cells (Fig. 5C).

To confirm that Ythdc2-170aa and Ythdc2 target STING, we investigated the interaction between Ythdc2-170aa and STING or Ythdc2 and STING. When cotransfected into MKC cells, Flag-tagged Ythdc2 (Flag-Ythdc2) coimmunoprecipitated with Myc-tagged STING (Myc-STING), and the Flag-tagged Ythdc2-170aa (Linear-FLAG-Ythdc2-170aa) also coimmunoprecipitated with Myc-STING (Fig. 5D). Moreover, compared with Ythdc2, Ythdc2-170aa has stronger binding ability with STING (Fig. 5D). Since Ythdc2 and Ythdc2-170aa share a common domain HELICc, we want to know whether the HELICc domain plays a role in binding to STING. Therefore, we constructed the HCLICc domain deletion mutant of the Ythdc2 (Flag-Ythdc2-△HELICc) plasmid. Immunocoprecipitation assay showed that the binding ability of STING to Ythdc2 decreased significantly after the HELICc domain’s deletion. Then, we cotransfected the Flag-Ythdc2 or Linear-FLAG-Ythdc2-170aa plasmids with Myc-STING plasmid and HA-ubiquitin plasmid (Fig. 5E).

To further explore which region of STING Ythdc2-170aa and Ythdc2 mainly bind to, we constructed the STING deletion mutant plasmid (Fig. 5F). Immunocoprecipitation assay showed that Ythdc2 mainly binds to the region between amino acids 1 and 157 of STING protein (Fig. 5G), and Ythdc2-170aa also mainly binds to the region between amino acids 42 and 157 of STING protein (Fig. 5H). Coimmunoprecipitation experiments showed that STING ubiquitination was markedly increased both in the presence of Ythdc2 and Ythdc2-170aa expression plasmid (Fig. 5I). Then, we tested whether Ythdc2 without HELICc domain could continue to promote the ubiquitination modification of STING. The results showed that Ythdc2-△HELICc could also promote the ubiquitination of STING, but the ubiquitination effect was greatly reduced compared with Ythdc2 (Fig. 5J). To further investigate Ythdc2 and Ythdc2-170aa-mediated STING ubiquitination, we used mutants in which all lysine residues except K48 or K11 were replaced with arginine (HA-ubiquitin-K48 and HA-ubiquitin-K11). Ythdc2 and Ythdc2-170aa-mediated ubiquitination of STING was significantly increased in the presence of wild-type HA-ubiquitin (HA-ubiquitin-WT) or HA-ubiquitin-K48 or HA-ubiquitin-K11 (Fig. 5K). These data indicate that Ythdc2 and Ythdc2-170aa both mediate K48 and K11-linked ubiquitination, and the HELICc domain is the key to the interaction between Ythdc2 and STING.

### N6-Methyladenosine(m6A) modification mediates circYthdc2 translation polypeptides

**Fig. 6:**
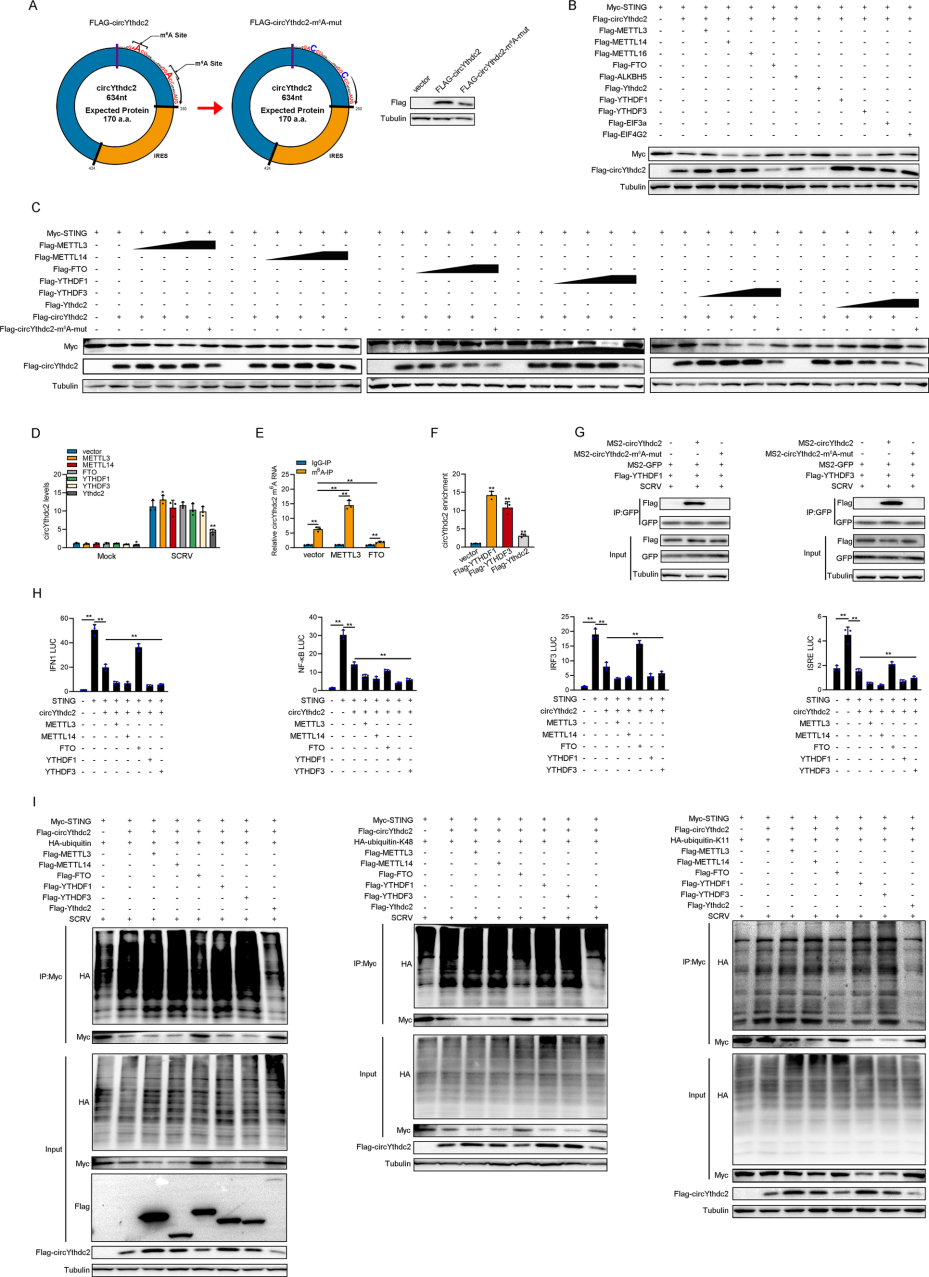
N6-methyladenosine modification mediates circYthdc2 translation polypeptides. A Upper panel: Schematic diagram of Flag-circYthdc2-m^6^A-mut plasmid construction. Lower panel: MKC cells were transfected with Flag-circYthdc2 or Flag-circYthdc2-m^6^A-mut plasmids, after 48 h, the immunoblot analysis was performed. **B** Myc-STING and Flag-circYthdc2 were cotransfected into MKC cells with m^6^A modification-related genes, respectively, and then the protein levels of Myc-STING and Flag-circYthdc2 were detected. **C** Left panel: Myc-STING and Flag-circYthdc2 were cotransfected into MKC cells with METTL3 or METTL14, respectively, and then the protein levels of Myc-STING and Flag-circYthdc2 were detected. Middle panel: Myc-STING and Flag-circYthdc2 were cotransfected into MKC cells with FTO or YTHDF1, respectively, and then the protein levels of Myc-STING and Flag-circYthdc2 were detected. Right panel: Myc-STING and Flag-circYthdc2 were cotransfected into MKC cells with YTHDF3 or Ythdc2, respectively, and then the protein levels of Myc-STING and Flag-circYthdc2 were detected. **D** Relative RNA levels of circYthdc2 in MKC cells after transfected with pcDNA3.1, METTL3, METTL14, YTHDF1, YTHDF3, FTO, and Ythdc2, respectively. **E** The m^6^A level alteration of circYthdc2 upon METTL3 or FTO overexpression was examined by MeRIP-qPCR. MKC cells were transfected with vector or METTL3 or FTO plasmid for 48 h. **F** The level of circYthdc2 upon YTHDF1 or YTHDF3 or Ythdc2 overexpression were examined by RIP-qPCR. MKC cells were transfected with vector or YTHDF1 or YTHDF3 or Ythdc2 plasmid for 48 h. **G** The protein level of YTHDF1 or YTHDF3 or Ythdc2 was examined by RNA pulldown. MKC cells were transfected MS2-GFP, MS2-circYthdc2 or MS2-circYthdc2-m^6^A-mut with vector or YTHDF1 or YTHDF3 or Ythdc2 plasmid for 48 h. **H** Relative luciferase activities were detected in MKC after cotransfection with STING expression plasmid, pRL-TK *Renilla* luciferase plasmid, luciferase reporters, circYthdc2, METTL3, METTL14, FTO, YTHDF1, YTHDF3. **I** Coimmunoprecipitation analysis of STING ubiquitination in MKC cotransfected with Myc-STING, Flag-circYthdc2 or m^6^A modification-related genes expression plasmid and HA-ubiquitin-WT, HA-ubiquitin-K11 or HA-ubiquitin-K48 plasmids. All data represented the mean ± SE from three independent triplicated experiments. *, *p* < 0.05; **, *p* < 0.01

Current research shows that there are two main ways to mediate circRNA translation, one is mediated by IRES and the other is mediated by m^6^A modification. Since Ythdc2, the host gene of circYthdc2, is an m^6^A-modified read protein, it is difficult not to associate whether there is m^6^A modification on circYthdc2. Then, by prediction, we found two potential m^6^A modification sites in the UTR region of circYthdc2. Therefore, we constructed the two potential m^6^A site mutant plasmids of circYthdc2 (Fig. 6A). Then, we transfected the wild-type and m^6^A site mutant FLAG-circYthdc2 plasmids into MKC cells, respectively. The results showed that the circYthdc2-170aa polypeptide expressed by the wild-type FLAG-circYthdc2 plasmid was significantly higher than that of the m^6^A site mutant FLAG-circYthdc2 plasmid. This result suggests that m^6^A modification may indeed exist on circYthdc2. We constructed some plasmids of m^6^A modification-related genes, including METTL3, METTL14, METTL16, FTO, ALKBH5, YTHDF1, YTHDF3, EIF3A, and EIF4G2. Subsequently, cotransfection experiments were conducted to verify whether these m^6^A modification-related genes could affect the translation efficiency of circYthdc2. As shown in Fig. 6B, the results indicated that METTL3, METTL14, YTHDF1, YTHDF3, and EIF4G2 could significantly increase the expression of circYthdc2-170aa polypeptide and significantly reduce the protein level of STING. It was also found that FTO could significantly reduce the expression of circYthdc2-170aa polypeptide and significantly increase the protein level of STING. To further verify the above experimental results, we conducted a concentration gradient experiment. The experimental results showed that METTL3, METTL14, YHTDF1, and YTHDF3 could significantly promote the expression of polypeptides in wild-type FLAG-circYthdc2 plasmids, but could not promote the expression of polypeptides in m^6^A site mutant plasmids (Fig. 6C).

The expression of circYthdc2-170aa polypeptides was closely related to the level of STING protein, when the expression of circYthdc2-170aa polypeptides increased, STING would correspondingly decrease, significant negative correlation; while FTO and Ythdc2 could significantly inhibit the expression of polypeptide in wild-type circYthdc2 plasmid. Notably, the expression of STING will be significantly decreased when transfected with Ythdc2 or circYthdc2 alone, but the expression of STING will be significantly increased when cotransfected with Ythdc2 and circYthdc2 (Fig. 6C). Therefore, we suspect that when the RNA level or polypeptide level of circYthdc2 rises, Ythdc2 will preferentially act on circYthdc2 instead of promoting the degradation of STING. Then, we tested the effects of METTL3, METTL14, FTO, YTHDF1, YTHDF3, and Ythdc2 on the RNA level of circYthdc2. The results showed that METTL3, METTL14, FTO, YTHDF1 and YTHDF3 had no significant effect on the RNA level of circYthdc2, but we found Ythdc2 could significantly reduce the RNA level of circYthdc2 (Fig. 6D). Furthermore, we explored its effect on the methylation level of circYthdc2 by performing a MeRIP-qPCR assay. Our results showed that overexpression of METTL3 dramatically increased the m^6^A level of circYthdc2 mRNA (Fig. 6E); in contrast, the m^6^A levels of circYthdc2 were significantly decreased after overexpression of FTO (Fig. 6E). To further verify whether YTHDF1, YTHDF3 and Ythdc2 can directly interact with circYthdc2, we conducted relevant RNA immunoprecipitation experiments. The experimental results showed that YTHDF1, YTHDF3, and Ythdc2 can significantly enrich circYthdc2, of which YTHDF1 has the highest enrichment efficiency, while Ythdc2 has a lower enrichment efficiency (Fig. 6F). At the same time, we also conducted RNA pulldown experiments. The results showed that wild-type circYthdc2 could enrich YTHDF1, YTHDF3 proteins, while m^6^A site mutant circYthdc2 could not enrich YTHDF1, and YTHDF3 proteins (Fig. 6G). Based on the above results, we believe that circYthdc2 can mediate the translation of polypeptides through m^6^A modification. METTL3 and METTL14 can act as “writer” proteins to promote the m^6^A modification of circYthdc2, FTO can act as “eraser” proteins to demethylate circYthdc2, and YTHDF1 and YTHDF3 can act as “reader” proteins to promote the translation of circYthdc2 into polypeptides, while Ythdc2 can act as a “reader” protein to promote the RNA degradation of circYthdc2.

We examined the effects of METTL3, METTL14, FTO, YTHDF1, and YTHDF3 on the NF-κB and IRF3 pathways. The results showed that METTL3, METTL14, YTHDF1, and YTHDF3 were roughly the NF-κB and IRF3 pathways that inhibited the activation of STING, while FTO was the NF-κB and IRF3 pathway that promoted the activation of STING (Fig. 6H). To further verify the above results, we cotransfected METTL3, METTL14, FTO, YTHDF1, YTHDF3, Ythdc2, and circythdc2 into MKC cells, respectively, and then detected the ubiquitination level of STING protein. The results were basically consistent with expectations. METTL3, METTL14, YTHDF1, and YTHDF3 could significantly promote the ubiquitination level of STING protein, while FTO and Ythdc2 could significantly inhibit the ubiquitination level of STING protein (Fig. 6I).

### Ythdc2-170aa, the translation product of circYthdc2, which is highly conserved in structure and function in vertebrates

**Fig. 7:**
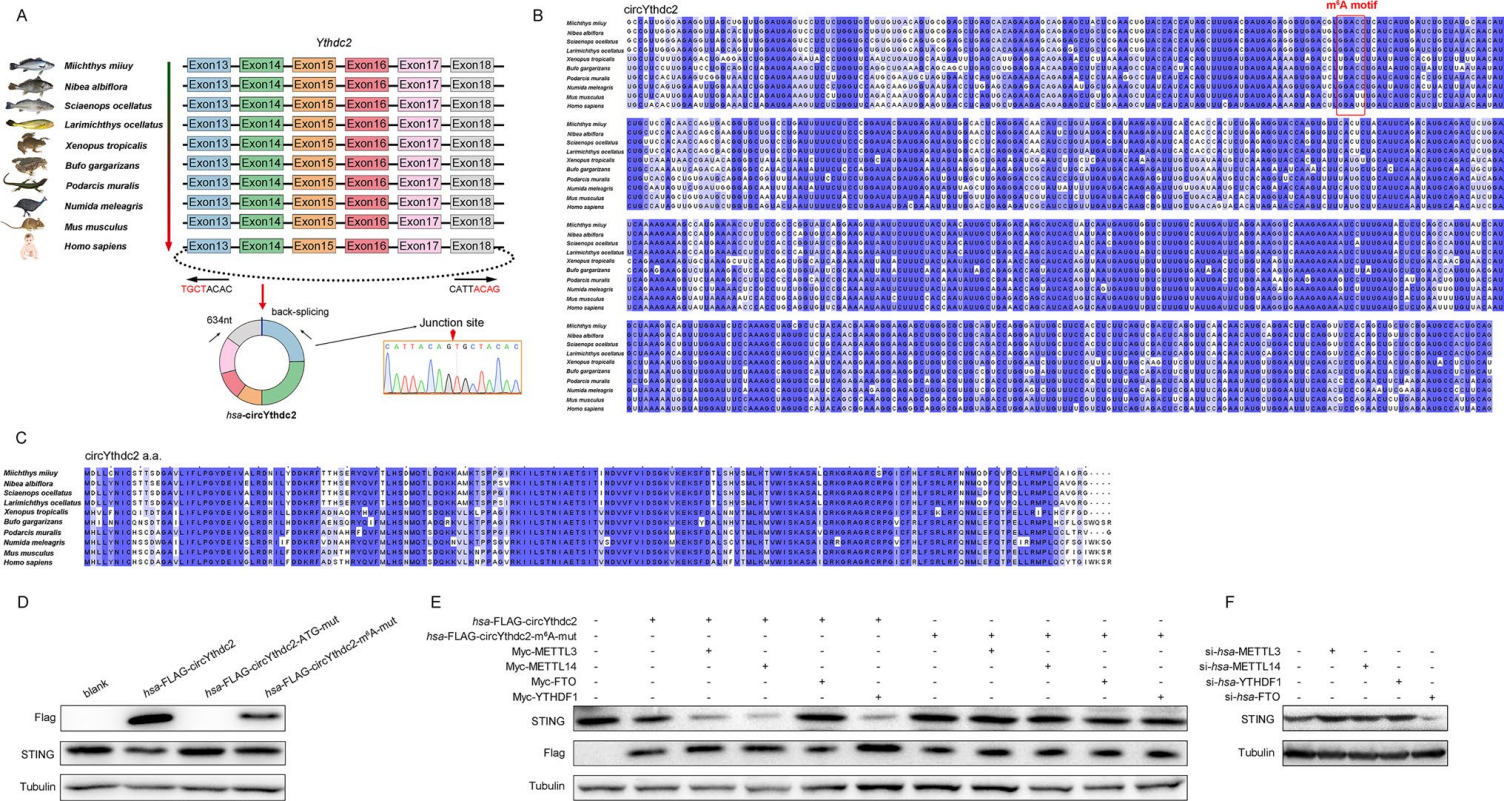
Ythdc2-170aa is highly conserved in structure and function in vertebrates. **A** CircYthdc2 exists in *Miichthys miiuy*, *Nibea albiflora*, *Sciaenops ocellatus*, *Larimichthys ocellatus*, *Xenopus tropicalis* (GenBank accession no. XM_031893156.1), *Bufo gargarizans* (GenBank accession no. XM_044275926.1), *Podarcis muralis* (GenBank accession no. XM_028748342.1), *Numida meleagris* (GenBank accession no. XM_021380759.1), *Mus musculus* (GenBank accession no. NM_001163013.1), *Homo sapiens* (GenBank accession no. NM_022828.5), and is composed of exon 13 to exon 18, with a length of 634nt. We confirmed the head-to-tail splicing of *hsa*-circYthdc2 in the *hsa*-circYthdc2 RT-PCR product by Sanger sequencing. **B** Sequence alignment of circYthdc2 from teleost fish to mammals. **C** Amino acid sequence alignment of circYthdc2 translated polypeptides from teleost fish to mammals. **D** HEK293 cells were transfected with vector or *hsa*-Flag-circYthdc2, *hsa*-Flag-circYthdc2-ATG-mut, *hsa*-Flag-circYthdc2-m^6^A-mut plasmids, after 48 h, the immunoblot analysis was performed. **E** HEK293 cells were transfected STING and *hsa*-Flag-circYthdc2 and *hsa*-Flag-circYthdc2-m^6^A-mut with METTL3 or METTL14 or YTHDF1 or FTO plasmids, after 48 h, the immunoblot analysis was performed. **F** HEK293 cells were transfected STING and *hsa*-Flag-circYthdc2 with si-*has*-METTL3 or si-*has*-METTL14 or si-*has*-YTHDF1 or si-*has*-FTO, after 48 h, the immunoblot analysis was performed. All data represented the three independent triplicated experiments

Through sequence alignment, we found that the sequence of exons 13 to 18 of Ythdc2 is highly conserved in amphibians, reptiles, birds, and mammals (including humans), all with a sequence length of 634 nt (Fig. 7A). Through further prediction, we speculate that exons 13 to 18 of Ythdc2 in these vertebrates can be reverse spliced to form circRNAs that can translate polypeptides. To confirm the objective existence of humans, *Homo sapiens* circYthdc2 (*hsa*-circYthdc2). First, *hsa*-circYthdc2 divergent primers were designed for RT-PCR amplification, and the amplified products were subjected to Sanger sequencing to confirm that *hsa*-circYthdc2 was spliced from the head to the tail (right panel of Fig. 7A). Through prediction, we found that the sequence of circYthdc2 is not only highly conserved in other vertebrates, but also may have a very conserved m^6^A modification site (Fig. 7B). Further studies found that circYthdc2 in different vertebrates may be able to translate polypeptides. We aligned the predicted amino acid sequences of circYthdc2 polypeptide, and the alignment results showed that the amino acid sequences of these polypeptides were highly conserved in vertebrates (Fig. 7C). To verify the correctness of the above prediction results, we constructed *hsa*-FLAG-circYthdc2, *hsa*-FLAG-circYthdc2-ATG-mut, and *hsa*-FLAG-circYthdc2-m^6^A-mut plasmids. Subsequently, the constructed plasmids were transfected into 293 cells, respectively, and the result showed that *hsa*-FLAG-circYthdc2 and *hsa*-FLAG-circYthdc2-m^6^A-mut could indeed be translated into polypeptide with about 24KD, while *hsa*-FLAG-circYthdc2-ATG-mut could not, and the expression level of the polypeptide translated by *hsa*-FLAG-circYthdc2 was much higher than that of *hsa*-FLAG-circYthdc2-m^6^A-mut. In addition, we also found that both *hsa*-FLAG-circYthdc2 and *hsa*-FLAG-circYthdc2-m^6^A-mut can promote the reduction of the level of human STING protein, while *hsa*-FLAG-circYthdc2-ATG-mut has no such effect (Fig. 7D). To further confirm that it can be driven by m^6^A modification, cotransfection experiments were conducted. As shown in Fig. 7E, the results indicated that METTL3, METTL14, and YTHDF1 could significantly promote the translation of polypeptide by *hsa*-FLAG-circYthdc2 plasmid and significantly reduce the protein level of STING, but not *hsa*-FLAG-circYthdc2-m^6^A-mut plasmid. It was also found that FTO could significantly reduce the translation of polypeptide by *hsa*-FLAG-circYthdc2 plasmid and significantly increase the protein level of STING, but not *hsa*-FLAG-circYthdc2-m^6^A-mut plasmid. In addition, we also found that silencing METTL3, METTL14, and YTHDF1 significantly increased the expression level of STING protein, while silencing FTO significantly reduced the expression level of STING protein (Fig. 7F). This result undoubtedly further confirms that circYthdc2 exists in humans and can be translated into polypeptide, and the inhibitory effect of its polypeptide on STING protein is similar to our confirmed results in teleost fish. Based on the above results, we believe that circYthdc2 is a highly conserved circRNA in vertebrates, and its translated polypeptides are also highly conserved. In addition, Ythdc2-170aa polypeptide has similar conserved functions both in fish and humans. The function of such a highly conserved circRNA must be of great significance.

## Discussion

At present, several types of ncRNAs, including lncRNA, pri-miRNA, and circRNA, have been reported to encode active polypeptides, which is undoubtedly a great discovery [30–32]. Compared with the mRNA responsible for coding which accounts for only 2% of the transcriptome, the proportion of ncRNA in the whole transcriptome is undoubtedly very huge. Even if only part of ncRNA can be translated into polypeptides, the number of polypeptides that may be translated is incalculable. In other words, there may be many active polypeptides that have not been found. However, it is not known whether this way of encoding polypeptides through ncRNA exists only in higher mammals such as mice and humans, and it is not clear whether such encoding polypeptides exist in lower vertebrates or even invertebrates. Among them, teleost is a representative population of lower vertebrates and an important part of early vertebrate evolution, so it is considered an excellent animal model in biological research. Therefore, it is very important to study whether ncRNA can encode active polypeptide in teleost fish because it is of great significance for a comprehensive understanding of ncRNA. In this study, we proved that circRNAs in teleost fish can also translate active polypeptides. We found a circRNA generated by the Ythdc2 linear gene, named circYthdc2, which can encode a polypeptide of 170 amino acids and participate in the regulation of antiviral innate immune response in teleost fish. Subsequently, through prediction, we found that circYthdc2 exists in reptiles, birds, amphibians, and mammals, and all of them are composed of exon 13 to exon 18 of the Ythdc2 gene. Moreover, the circYthdc2 sequence of these species is highly conserved, with a length of 634nt. Further prediction results show that circYthdc2 in reptiles, birds, amphibians, and mammals can translate polypeptides, and the amino acid sequence of its polypeptide is highly conserved. Therefore, we first confirmed that circYthdc2 exists in humans by Sanger sequencing, and then we constructed a human circYthdc2 overexpression plasmid for expression verification. The results showed that human circYthdc2 can also encode the same polypeptide and play a similar function to that of teleost circYthdc2. All the above results show that circYthdc2 may play an indispensable role in vertebrates, and it is of great significance to explore its function. In addition, this discovery confirms for the first time that the ability of circRNA to encode functional proteins is evolutionarily conservative, which undoubtedly fills the gap in the function of circRNA-encoded proteins from lower vertebrates to higher lactation, and makes people’s understanding of circRNA more systematic and comprehensive.

**Fig. 8:**
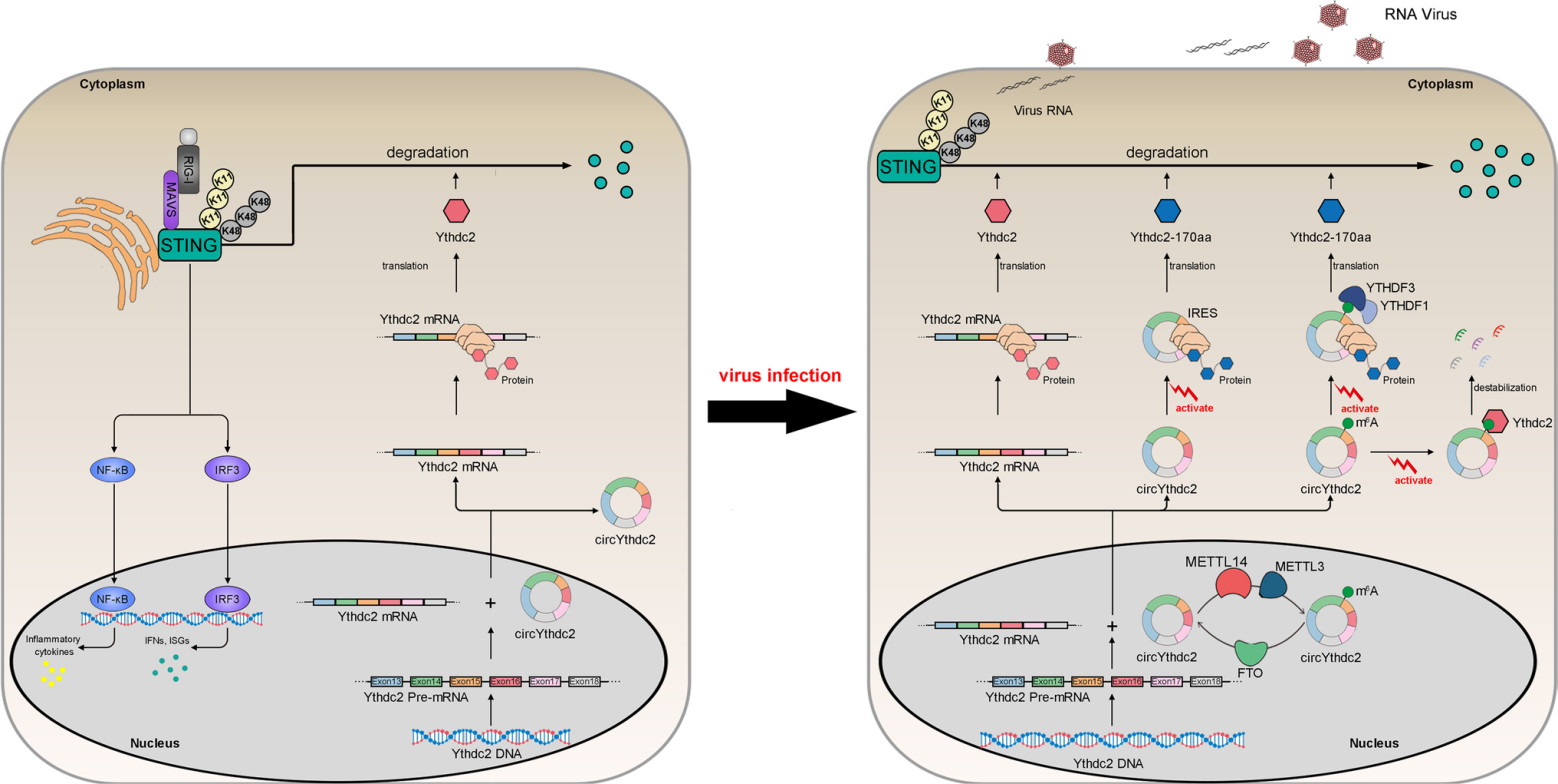
Schematic diagram of the mechanism underlying Ythdc2-170aa and Ythdc2 both promoted K11 and K48-linked ubiquitination of STING. Under normal circumstances, circYthdc2 does not translate to produce polypeptides. When SCRV virus infects the host, the pathway of circYthdc2 translating polypeptides is activated. There are two pathways for circYthdc2 to be translated into polypeptides, the one is IRES-mediated translation pathway and another m^6^A modification mediated translation pathway. In addition, Ythdc2 will preferentially promote the RNA degradation of circYthdc2 when circYthdc2 is produced in large quantities. Ythdc2-170aa and Ythdc2 both could promote the STING protein degradation and represses STING-mediated antiviral responses, thereby regulating viral replication. Ythdc2-170aa and Ythdc2 both promoted K11 and K48-linked ubiquitination of STING, thereby inhibited the antiviral responses and help the virus escape

In addition, it is puzzling that the circRNA does not have the 5'-hat structure and 3'-poly (A) tail structure necessary for traditional translation [33–35]. Through in-depth research, it is found that there are other translation methods. Current research shows that there are two main forms of how circRNA translates polypeptides. One is to promote the combination of initiation factor or ribosome with circRNA through the IRES element sequence, which leads to the beginning of translation [36, 37]. For example, circ-FBXW7 and circ-AKT3 induce the translation of polypeptides through IRES element sequences and play corresponding functions [12, 14]. Another form is through N6-methyladenosine(m^6^A) modification of circRNA, and m^6^A-modified circRNA can effectively start translation [25]. However, it is still unclear under what conditions the translation pathway mediated by m^6^A modification is conducted. In addition, it is also unclear whether IRES and m^6^A modification co-mediate circRNA translation. If IRES and m^6^A modification co-mediate circRNA translation, it is worth exploring under what conditions which translation mode is initiated. In this study, our results provided direct evidence the novel circRNA circYthdc2, which contains an open reading frame (ORF) both driven by the IRES sequence or m^6^A site, and encode a functional polypeptide of 170 amino acids, can inhibit the antiviral immune response and promote the escape of SCRV virus, and we named this polypeptide Ythdc2-170aa. Further studies show that METTL3 and METTL14 act as “writers” to perform m^6^A modification on circYthdc2, while FTO acts as “eraser” to demethylate circYthdc2, and YTHDF1 and YTHDF3 act as “readers” to jointly promote the translation of polypeptides by circYthdc2. We found that Ythdc2-170aa has only one domain HELICc, and this domain has the same amino acid sequence as the HELICc domain of Ythdc2 protein. Ythdc2 can also target and bind STING and also inhibit STING-mediated antiviral immune response. Ythdc2-170aa and Ythdc2 can promote the ubiquitination of K11 and K48 link of STING through targeted binding to STING, to promote the degradation of STING protein and weaken the antiviral immune response caused by SCRV viral, and finally lead to the escape of SCRV virus (Fig. 8). In addition, these polypeptides encoded by circRNA often have a strong relationship with the host gene of circRNA. For example, the polypeptide AKT-174aa encoded by circ-AKT3 can prevent the phosphorylation of the host gene AKT3 of circ-AKT3 by acting as a molecular bait [14]. Moreover, circβ-catenin can encode a polypeptide that can promote its host gene β-catenin phosphorylation and degradation [37]. circ-SHPRH can encode a polypeptide SHPRH-146aa, which can inhibit the ubiquitination and degradation of its host gene SHPRH protein [25]. In addition, circLgr4 can encode a polypeptide, which can bind with its host gene LGR4 protein and promote the Wnt/β-Catenin signal pathway [38]. Since many host genes that produce circRNA have protein-coding ability and are functional genes with important significance in life activities, therefore, it is of great significance to discover and reveal this type of circRNA that can regulate the host gene. Ythdc2 is an important “read” protein involved in m^6^A modification [39, 40], this undoubtedly provides a certain reference for exploring the relationship between Ythdc2 and circYthdc2. The experimental results showed that Ythdc2 does not participate in the translation process of circYthdc2, but it can degrade circYthdc2 by acting as an m^6^A “read”. When circYthdc2 is abundant, Ythdc2 preferentially degrades circYthdc2 and no longer promotes the degradation of STING. We believe that this may be a self-protection mechanism of the host. When the virus infects the host, the expression of circYthdc2 increases, which promotes the degradation of STING and weakens the antiviral immune response of the host. However, when circYthdc2 is expressed in large quantities, Ythdc2 preferentially starts to promote the degradation of circYthdc2, which further inhibits the degradation of STING and stabilizes the antiviral immune response of the host. This is undoubtedly an arms race between the host defense mechanism and the virus hijacking mechanism.

## Methods

### Ethics statement

All animal experimental procedures were performed in accordance with the National Institutes of Health’s Guide for the Care and Use of Laboratory Animals, and the experimental protocols were approved by the Research Ethics Committee of Shanghai Ocean University (No. SHOU-DW-2018-047).

### Sample and challenge

Miiuy croaker (∼50 g) was obtained from Zhoushan Fisheries Research Institute, Zhejiang Province, China. Fish was acclimated in aerated seawater tanks at 25 °C for 6 weeks before experiments. Experimental procedures for SCRV infection were performed as described [41].

### Sequencing analysis and circRNAs identification

The spleen tissues from three healthy fishes and three SCRV-challenged fishes were separated and total RNAs were extracted for the construction of the cDNA library. Afterwards, the cDNA libraries were sequenced using Illumina HiSeq 2500 platform. The sequencing data have been deposited in the Sequence Read Archive (SRA) at the National Center for Biotechnology Information (NCBI) under accession number PRJNA685924. Clean reads were aligned against the *M. miiuy* reference genome using the mapping program TopHat2 [42]. The unmapped reads were extracted and further aligned with *M. miiuy* reference sequence by TopHat-fusion software [43]. The junction reads with noncolinear ordering alignment on the same chromosome were regarded as candidate back-spliced junction reads. The back-spliced junction reads were used for the identification of circRNAs by CIRI software [44].

### Cell culture and treatment

MKC, MSpC, MIC, MBrC, MMC, and MLC were cultured in L-15 medium (HyClone) supplemented with 15% fetal bovine serum (FBS; Gibco), 100 U/ml penicillin, and 100 μg/ml streptomycin at 26 °C [41]. Epithelioma papulosum cyprini cells (EPC) were maintained in medium 199 medium (Invitrogen) supplemented with 10% FBS, 100 U/ml penicillin, and 100 mg/ml streptomycin at 28 °C in 5% CO_2_ [41]. HEK293 cells were cultured in DMEM medium which contained the 10% FBS, 2 mM l-glutamine, 100 U/ml penicillin, and 100 mg/ml streptomycin, and under humidified conditions with 5% CO_2_ at 37 °C [45]. For stimulation experiments, MKC and MIC cells were challenged with SCRV at a multiplicity of infection (MOI) of 5 and harvested at different times for RNA extraction [41].

### Plasmids construction

To construct the Luc-circYthdc2-IRES-WT, Luc-circYthdc2-IRES-DEL1, Luc-circYthdc2-IRES-DEL2 reporter vector, the IRES region of *M. miiuy* circYthdc2, as well as the base 1 to 87 in IRES region of circYthdc2 or the base 88 to 174 in IRES region of circYthdc2, were amplified using PCR and cloned into Luc2-IRES-report luciferase reporter vector (Geneseed Biotech). Meanwhile, the IRES region of *M. miiuy* circYthdc2, as well as the base 1 to 87 in the IRES region of circYthdc2 or the base 88 to 174 in the IRES region of circYthdc2 were inserted into the pEGFP vector (Invitrogen), which included the sequence of enhanced GFP. To construct the circYthdc2 overexpression vector and FLAG-circYthdc2 overexpression vector, the full-length circYthdc2 cDNA was amplified by specific primer pairs and cloned into pLC5-circ vector (Geneseed Biotech), which contained a front and back circular frame to promote RNA circularization. The full length of circYthdc2 cDNA and FLAG sequence were amplified by specific primer pairs and cloned into pLC5-circ vector (Geneseed Biotech), which do not contain a back circular frame to promote RNA circularization. The full length of the Ythdc2-170aa CDS region and FLAG sequence was amplified by specific primer pairs and cloned into the pcDNA3.1 vector (Invitrogen). The STING, MAVS, TRIF, and TBK1 overexpression plasmid was constructed as described [41, 45, 46]. The sequences of all primers are listed in Table S1.

### RNA oligoribonucleotides

The RNA interferences for circYthdc2 are as follows: si-circYthdc2-1 sequence was 5′-GAUGCCACUGCAGGCCAUUTT-3′. In addition, the si-circYthdc2-2 sequence was 5′-CCACUGCAGGCCAUUGGGATT-3; the scrambled control RNA sequences were 5′-GAUGCCACUGCAGUAACGGTT-3′. The RNA interference for Ythdc2 is as follows: si-Ythdc2 sequence was 5′-GGAAAGGACUACAACGCUUTT-3′. The RNA interference for *hsa*-METTL3 is as follows: si-*hsa*-METTL3 sequence was 5′-CAGGAGAUCCUAGAGCUAUTT-3′. The RNA interference for *hsa*-METTL14 is as follows: si-*hsa*-METTL14 sequence was 5′-GAUAGCACUGUGGUGUGGATT-3′. The RNA interference for *hsa*-YTHDF1 is as follows: si-*hsa*-YTHDF1 sequence was 5′-GGAACAACAUCUAUCAGCATT-3′. The RNA interference for *hsa*-FTO is as follows: si-*hsa*-FTO sequence was 5′-GGUUCACAACCUCGGUUUATT-3′. The scrambled control RNA sequences were 5′-UUCUCCGAACGUGUCACGUTT-3′.

### Cell transfection

Transient transfection of cells with miRNA mimic, miRNA inhibitor, or siRNA was performed in 24-well plates using Lipofectamine RNAiMAX (Invitrogen), and cells were transfected with DNA plasmids were performed using Lipofectamine 3000 (Invitrogen) according to the manufacturer’s instructions. For functional analyses, the overexpression plasmid (500 ng per well) or control vector (500 ng per well) and siRNA (100 nM) were transfected into cells in culture medium and then harvested for further detection. For luciferase experiments, pmirGLO or Luc2-IRES-report (500 ng per well) containing the plasmid of circYthdc-170aa were transfected into cells.

### RNA extract and quantitative real-time PCR

For the isolation and purification of both cytoplasmic and nuclear RNA from MKC and MIC cells, the Cytoplasmic & Nuclear RNA Purification Kit has been used according to the manufacturer’s instructions (Norgen Biotek). Total RNA was isolated with TRIzol Reagent (Invitrogen) and the cDNA was synthesized using the FastQuant RT Kit (Tiangen) which includes DNase treatment of RNA to eliminate genomic contamination. The expression patterns of each gene were performed using SYBR Premix Ex TaqTM (Takara). Real-time PCR was performed in an Applied Biosystems® QuantStudio 3 (Thermo Fisher Scientific). GAPDH was employed as endogenous controls for mRNA, respectively. Primer sequences are displayed in Table S1.

### Luciferase report assay

The Luc2-IRES-report, Luc2-circYthdc2-IRES-WT, Luc2-circYthdc2-IRES-DEL1, and Luc2-circYthdc2-IRES-DEL2 were transfected into EPC cells, respectively. At 48 h post-transfection, reporter luciferase activities were measured using the dual-luciferase reporter assay system (Promega). To determine the functional regulation of Ythdc2-170aa, cells were cotransfected the overexpression plasmid of STING, MAVS, TRIF, or TBK1 and Ythdc2-170aa overexpression plasmid, together with IFN1, NF-κB, IRF3, and IRSE luciferase reporter gene plasmids, phRL-TK Renilla luciferase plasmid, negative controls. At 48 h post-transfection, the cells were lysed for reporter activity using the dual-luciferase reporter assay system (Promega). STING overexpression plasmid and Ythdc2 overexpression plasmid, together with IFN1, NF-κB, IRF3, and IRSE luciferase reporter gene plasmids, phRL-TK *renilla* luciferase plasmid, negative controls. At 48 h post-transfection, the cells were lysed for reporter activity. All the luciferase activity values were achieved against the *renilla* luciferase control. Transfection of each construct was performed in triplicate in each assay. Ratios of *renilla* luciferase readings to firefly luciferase readings were taken for each experiment, and triplicates were averaged.

#### Antibody generation and Western blotting

A polyclonal antibody against the Ythdc2-170aa polypeptide produced by circYthdc2 was obtained by inoculating rabbits (GenScript). Cellular lysates were generated using 1 × SDS-PAGE loading buffer. Proteins were extracted from cells and measured with the BCA Protein Assay kit (Vazyme), then subjected to SDS-PAGE (8%) gel and transferred to PVDF (Millipore) membranes by semidry blotting (Bio-Rad Trans Blot Turbo System). The membranes were blocked with 5% BSA. Protein was blotted with different antibodies. The antibody against STING was diluted at 1: 500 (Abcam); The antibody against Ythdc2 was diluted at 1: 500 (Abcam); the antibody against Ythdc2-170aa was diluted at 1: 200 (GenScript); anti-Flag, anti-HA, anti-Myc, and anti-Tubulin monoclonal antibody were diluted at 1: 2,000 (Sigma); and HRP-conjugated anti-rabbit IgG or anti-mouse IgG (Abbkine) at 1: 5,000. The results were representative of three independent experiments. The immunoreactive proteins were detected using WesternBright™ ECL (Advansta). The digital imaging was performed with a cold CCD camera.

#### RNase R and actinomycin D treatment

For RNase treatment, the RNAs (10 μg) from MBrC and MIC cells were treated with RNase R (3 U/μg, Epicenter) and incubated for 30 min at 37 °C. For actinomycin D treatment, MBrC and MIC cells were treated with 5 μg/ml actinomycin D (Sigma) and collected in a series of time intervals. Then, the expression of circSamd4a and the linear mRNA was detected by qRT-PCR.

#### Nucleic acid electrophoresis

The cDNA and gDNA PCR products were investigated using 2% agarose gel electrophoresis with TAE running buffer. DNA was separated by electrophoresis at 100 V for 30 min. The DNA marker was the Super DNA Marker (CWBIO). The bands were examined by UV irradiation.

#### Immunoprecipitation assay

For immunoprecipitation (IP) experiments, MKC cells, EPC cells, and HEK293 cells were seeded onto 10 cm^2^ plate overnight then cotransfected with 5 μg indicated plasmids. At 48 h post-transfection, the cells were washed three times with ice-cold PBS. Then, the cells were lysed with 500 μl western and IP lysis buffer (Beyotime) containing protease inhibitor cocktail (Bitake) at 4 °C for 30 min on a rocker platform. Then, the cellular debris was removed by centrifugation at 14,000 g for 15 min at 4 °C. After centrifugation, the supernatant was transferred into a fresh centrifuge tube and incubated with 50 μl protein A + G (Sigma) together with 1 μg monoclonal anti-Flag (Sigma) overnight at 4 °C with constant and soft agitation. The following day, the IP protein was collected by centrifugation at 2500 g for 5 min at 4 °C. Then, beads were washed five times with western and IP lysis buffer and resuspended in 60 μl 2 × SDS loading buffer. The immunoprecipitates and whole-cell lysates (WCLs) were analyzed by immunoblotting.

#### Fluorescent microscopy

MKC cells were seeded onto 24-well plate and transfected using Lipofectamine 3000 (Invitrogen) with indicated plasmids for 48 h. Fluorescence signals were assessed by confocal microscopy (Leica).

#### Cell viability

Cell viability was measured at 48 h after transfection in MKC and MIC cells with CellTiter-Glo Luminescent Cell Viability assays (Promega) according to the manufacturer’s instructions. These experiments were repeated for three times.

#### MeRIP-seq assays and sequencing data analysis

Total RNA was isolated using TRIzol reagent. m^6^A immunoprecipitation and library preparation were performed following the manufacturer’s protocol. Poly (A) RNA is purified from 50 μg total RNA using Dynabeads Oligo (dT) 25–61,005 (Thermo Fisher, CA, USA) using two rounds of purification. Then, the poly(A) RNA was fragmented into small pieces using Magnesium RNA Fragmentation Module (NEB, cat. e6150, USA) under 86 °C for 7 min. Then, the cleaved RNA fragments were incubated for 2 h at 4 °C with m^6^A-specific antibody (No. 202003, Synaptic Systems, Germany) in IP buffer (50 mM Tris–HCl, 750 mM NaCl and 0.5% Igepal CA-630). Then, the IP RNA was reverse-transcribed to create the cDNA by SuperScript™ II Reverse Transcriptase (Invitrogen, cat. 1896649, USA). The average insert size for the final cDNA library was 300 ± 50 bp. For sequencing data analysis, we used HISAT2 to map reads to the reference genome of *M. miiuy*. Mapped reads of IP and input libraries were provided for R package exomePeak, which identifies m^6^A peaks with bed or bigwig format that can be adapted for visualization on the IGV software. MEME and HOMER were used for de novo and known motif finding followed by localization of the motif with respect to peak summit. Called peaks were annotated by intersection with gene architecture using R package ChIPseeker. The differentially expressed mRNAs were selected with log2 (fold change) > 1 or log2 (fold change) < -1 and p value < 0.05 by R package edgeR.

#### RNA immunoprecipitation assay (RIP)

RIP experiments were performed using the Magna RIP RNA-Binding Protein Immunoprecipitation Kit (Millipore) following the manufacturer’s protocol. The RIP assay was also conducted in MKC cells (~ 2.0 × 10^7^) transfected with YTHDF1 and YTHDF3. After 48 h transfection, the MKC cells were used in RIP assays via the Magna RIP™ RNA-Binding Protein Immunoprecipitation Kit (Millipore) and an anti-flag antibody following the manufacturer’s protocol. RNA was extracted from the remaining beads and qPCR was used to evaluate the expression levels of circYthdc2.

#### RNA pulldown assay

The MS2-RNA pulldown assay was also conducted in MKC cells (~ 2.0 × 10^7^) transfected with pLC5-ciR-MS2, pLC5-ciR-MS2-circYthdc2, pLC5-ciR-MS2-circYthdc2-mut, YTHDF1, YTHDF3, or pMS2-GFP (Addgene). To construct plasmids that could produce circYthdc2 identified by the MS2 protein, an MS2 fragment was cloned into pLC5-ciR, pLC5-ciR-circYthdc2, and mutated type of circYthdc2 plasmid. Furthermore, a GFP and MS2 gene fusion expression plasmid was also constructed to produce a GFP-MS2 fusion protein that could bind with the MS2 fragment and be identified using an anti-GFP antibody (Abcam).

To conduct pulldown assay with MS2-lated circYthdc2, MKC cells was harvested at 48 h after transfection, then incubated on ice for 30 min in lysis buffer (20 mM Tris, pH 7.5, 200 mM NaCl, 2.5 mM MgCl_2_, 1 mM DTT, 60 U/ml Superase-In, 0.05% Igepal, protease inhibitors). The lysates were precleared by centrifugation for 5 min, and 50 μl of the sample was aliquoted for input. The remaining lysates were incubated with M-280 streptavidin magnetic beads (Sigma). To prevent non-specific binding of RNA and protein complexes, the beads were coated with RNase-free BSA and yeast tRNA (Sigma). The beads were incubated for 4 h at 4 °C, washed twice with ice-cold lysis buffer, three times with the low salt buffer (0.1% SDS, 1% Triton X-100, 2 mM EDTA, 20 mM Tris–HCl pH 8.0 and 150 mM NaCl) and once with the high salt buffer (0.1% SDS, 1% Triton X-100, 2 mM EDTA, 20 mM Tris–HCl pH 8.0 and 500 mM NaCl). Then, beads were washed five times with western and lysis buffer and resuspended in 60 μl 2 × SDS loading buffer.

#### Statistical analysis

Data are expressed as the mean ± SE from at least three independent triplicated experiments. Student’s *t* test was used to evaluate the data. The relative gene expression data were acquired using the 2^−∆∆CT^ method, and comparisons between groups were analyzed by one-way analysis of variance (ANOVA) followed by Duncan’s multiple comparison tests [47]. A value of *p* < 0.05 was considered significant.

### Supplementary Information

Below is the link to the electronic supplementary material.

Fig. S1. Expression profiles and characterization of circYthdc2. (A) The expression levels of Ythdc2 and circYthdc2 in spleen samples were measured by qRT-PCR at indicated time after SCRV infection and Poly (I:C) stimulation. (B) The expression levels of Ythdc2 and circYthdc2 in MKC cells were measured by qRT-PCR at indicated time after SCRV infection. (C) Relative expression of Ythdc2 and circYthdc2 in indicated cell lines was determined by qRT-PCR, including RNase R treated group and not treated group. (D) Actinomycin D treatment was applied to evaluate the stability of Ythdc2 and circYthdc2 mRNA in MKC and MIC cells. (E) circYthdc2 was mainly localized in the cytoplasm. RNA isolated from nuclear and cytoplasm was used to analyze the expression of circYthdc2 by RT-PCR; All data represent the means ± SE from three independent triplicate experiments. *, p < 0.05; **, p < 0.01. Supplementary file1 (DOCX 32 KB)

Supplementary file2 (TIF 832 KB)

## Data Availability

The data that support the findings of this study are available in the methods and/or supplementary material of this article.
