# Supplementary material for: CircYthdc2 generates polypeptides through two translation strategies to facilitate virus escape
Source: Cell Mol Life Sci. 2024 Feb 15;81(1):91. doi: 10.1007/s00018-024-05148-9 (PMC10869389; doi:10.1007/s00018-024-05148-9)
Supplement: Supplementary file 1 — Fig. S1. Expression profiles and characterization of circYthdc2. (A) The expression levels of Ythdc2 and circYthdc2 in spleen samples were measured by qRT-PCR at indicated time after SCRV infection and Poly (I:C) stimulation. (B) The expression levels of Ythdc2 and circYthdc2 in MKC cells were measured by qRT-PCR at indicated time after SCRV infection. (C) Relative expression of Ythdc2 and circYthdc2 in indicated cell lines was determined by qRT-PCR, including RNase R treated group and not treated group. (D) Actinomycin D treatment was applied to evaluate the stability of Ythdc2 and circYthdc2 mRNA in MKC and MIC cells. (E) circYthdc2 was mainly localized in the cytoplasm. RNA isolated from nuclear and cytoplasm was used to analyze the expression of circYthdc2 by RT-PCR; All data represent the means ± SE from three independent triplicate experiments. *, p < 0.05; **, p < 0.01. Supplementary file1 (DOCX 32 KB) [file 18_2024_5148_MOESM1_ESM.docx]

**Table S1** PCR primer information in this study.

| Primer | Sequences (5’-3’) |
| --- | --- |
| STING-qRT-F | AGGCACCAACAATTCCAG |
| STING-qRT-R | ACGGAGCAGGCTTCACTT |
| TNF-α-qRT-F | GTTTGCTTGGTACTGGAATGG |
| TNF-α-qRT-R | TGTGGGATGATGATCTGGTTG |
| IFN1-qRT-F | TACGATGGCTAATAACTCC |
| IFN1-qRT-R | CATTGACAAAGTGCTCCA |
| MX1-qRT-F | GCTGCTTGTTTACTCCCA |
| MX1-qRT-R | ACCTGCATCATCTCCCTC |
| ISG15-qRT-F | TGAACGGACAGAAGACGC |
| ISG15-qRT-R | TGAGGAATACCTGCATGG |
| Viperin-qRT-F | ACCCGTCCAAGTCCATAC |
| Viperin-qRT-R | TCATGTCAGCTTTGCTCC |
| SCRV-qRT-F | GGGCTGGATGATAGACGATTG |
| SCRV-qRT-R | TGGCGGAGGTGCTTGATATGG |
| circYthdc2-divergent-F | GATGCCACTGCAGGCCAT |
| circYthdc2-divergent-R | GTGGAGCAGATGTTGCATAGCA |
| circYthdc2-convergent-F | CCATCGCCTGTACTCTG |
| circYthdc2-convergent-R | GGTCGCTGAAGGTGTTA |
| GAPDH-qRT-F | ACCTTCACTCCTCCATCTT |
| GAPDH-qRT-R | AGGTCACAGACACGGTTG |
| circYthdc2-m^6^A-qRT-F | TCCACAGCTGCTGCGG |
| circYthdc2-m^6^A-qRT-R | CATGATGAGGTCCACG |
| circYthdc2-F | CGGAATTCTAATACTTTCAGGCCATTGGGAGAGGTTAGC |
| circYthdc2-R | CGGGATCCAGTTGTTCTTACCTGCAGTGGCATCCGCA |
| circYthdc2-FLAG-F | CGGAATTCTAATACTTTCAGGACGACGATAAGGCCATTGGGAGAGGTTAGC |
| circYthdc2-FLAG-R | CGGGATCCAGTTGTTCTTACATCCTTGTAATCCTGCAGTGGCATCCGCA |
| circYthdc2-FLAG-m^6^A-1F | CTACTCGCCCTGTACCACCATAGCTTTGACGATGA |
| circYthdc2-FLAG-m^6^A-1R | TGGTACAGGGCGAGTAGCTCCTGCTCTTCTGTGC |
| circYthdc2-FLAG-m^6^A-2F | TGGCCCTCATCATGGATCTGCTATGCAACATC |
| circYthdc2-FLAG-m^6^A-2R | ATCCATGATGAGGGCCACGTCCACCCTCTCATCGTCA |
| circYthdc2-FLAG-MS2-F | ACATGAGGATCACCCATGTCTGCAGCTGTTTGGATGAGTCCTCTCTGG |
| circYthdc2-FLAG-MS2-R | CATGGGTGATCCTCATGTTTTCTAGCTAACCTCTCCCAATGGCCTG |
| Linear-FL-Ythdc2-AG-F | CAGGTGGATCCTAGCTAACAACTCCATACTTTT |
| Linear-FL-Ythdc2-AG-R | TTAGCTAGGATCCACCTGCAGTGGCATCCGCAG |
| Ythdc2-170aa-FLAG-1F | GACGATGACGACAAGAAGCTTTGTTTGGATGAGTCCTCTCTGGT |
| Ythdc2-170aa-FLAG-1R | TGATGGATATCTGCAGAATTCCTAACCTCTCCCAATGGCCTGCAGTGGCATCCGCAG |
| Luc2-circYthdc2-IRES-WT-1F | AACGACCAGTAATAAGGTACCAAGAGATTCACCACCCACTCTGA |
| Luc2-circYthdc2-IRES-WT-1R | CTTAGCATCGGCCATGAATTCAATGACAAAGACCACATCATTGATAGT |
| Luc2-circYthdc2-IRES-DEL1-1F | AACGACCAGTAATAAGGTACCAAGAGATTCACCACCCACTCTGA |
| Luc2-circYthdc2-IRES- DEL1-1R | CTTAGCATCGGCCATGAATTCAATGACAAAGACCACATCATTGATAGT |
| Luc2-circYthdc2-IRES- DEL2-1F | AACGACCAGTAATAAGGTACCACCTCTCCGCCCGGTATCA |
| Luc2-circYthdc2-IRES- DEL2-1R | CTTAGCATCGGCCATGAATTCAATGACAAAGACCACATCATTGATAGT |
| circYthdc2-IRES-WT-GFP-1F | CTACCGGACTCAGATCTCGAGAAGAGATTCACCACCCACTCTGA |
| circYthdc2-IRES-WT-GFP-1R | GTACCGTCGACTGCAGAATTCAATGACAAAGACCACATCATTGATAGT |
| circYthdc2-IRES-DEL1-GFP-1F | CTACCGGACTCAGATCTCGAGAAGAGATTCACCACCCACTCTGA |
| circYthdc2-IRES- DEL1-GFP-1R | GTACCGTCGACTGCAGAATTCTTTCATGGCTTTCTTTTGATCCA |
| circYthdc2-IRES- DEL2-GFP-1F | CTACCGGACTCAGATCTCGAGACCTCTCCGCCCGGTATCA |
| circYthdc2-IRES- DEL2-GFP-1R | GTACCGTCGACTGCAGAATTCAATGACAAAGACCACATCATTGATAGT |
| STING-Myc-1F | ACTATAGGGAGACCCAAGCTTATGCTGTGCCTCCAGGATCA |
| STING-Myc-1R | TGATGGATATCTGCAGAATTCTTACATAAATCCCTGATAATTGTCGG |
| STING-Myc-ΔTM-1F | AACTCATCTCTGAAGAGGATCTGAAGCTTATGCTGTGCCTCCAGG |
| STING-Myc-ΔTM-1R | CCTCTTCAGAGATGAGTTTCTGCTCCATGGTCTCCCTATAGTGAGTCGTAT |
| STING-Myc-ΔN-1F | TCTGAAGAGGATCTGAAGCTTATGCTGTGCCTCCAGGATCA |
| STING-Myc-ΔN-1R | TGATGGATATCTGCAGAATTCTAACCTTTGAGGAGACAAAAACGC |
| STING-Myc-ΔC-1F | TCTGAAGAGGATCTGAAGCTTAAAGAGGAGTTCTGCCTCAACCC |
| STING-Myc-ΔC-1R | TGATGGATATCTGCAGAATTCTTACATAAATCCCTGATAATTGTCGG |
| METTL3-Flag-1F | CCCAAGCTTCTCGTCATGTCGGACACAT |
| METTL3-Flag-1R | CCGGAATTCTGCGGGGATCACATACAG |
| METTL14-Flag-1F | CCCAAGCTTACGAAGGAGAAAATGAACAGTC |
| METTL14-Flag-1R | CGCGGATCCTGTGATTGGAGATTCATAAGGC |
| METTL16-Flag-1F | CCCAAGCTTAACAAGGAAGACTCCAACA |
| METTL16-Flag-1R | CCGGAATTCCTTCTCAAAAGCTGTATGC |
| FTO-Flag-1F | CGGGGTACCCACAACTCCAGGAACATG |
| FTO-Flag-1R | TGCTCTAGAGCCTCTACATTAAGAAAAGC |
| ALKBH5-Flag-1F | CCCAAGCTTGGCTATCTGTCAGCTACTAC |
| ALKBH5-Flag-1R | CCGGAATTCCTTCGTCTGCCAGAAAC |
| YTHDF1-Flag-1F | CGGGGTACCCATTTCAACATGACCACCAA |
| YTHDF1-Flag-1R | CCGGAATTCGCAGCCGTCTTCTGTTTACT |
| YTHDF3-Flag-1F | CCCAAGCTTTCAGTGCAAAACGGATCAAT |
| YTHDF3-Flag-1R | CGCGGATCCGCCTTTCCTCCTTTGTGGTT |
| EIF3a-Flag-1F | GACGACAAGAAGCTTGGTACCATGCCGGCGTATTTTCAACG |
| EIF3a-Flag-1R | GTTCACGGACTGTCTTCTTCTTGATCTGCTCGTG |
| EIF3a-Flag-2F | GAAGAAGACAGTCCGTGAACGGCTGGAGC |
| EIF3a-Flag-2R | TGATGGATATCTGCAGAATTCTCAGCGGCGGACAGTGGT |
| EIF4G2-Flag-1F | GACGATGACGACAAGAAGCTTATGCTGGGCAACATCAAATTC |
| EIF4G2-Flag-1R | TGATGGATATCTGCAGAATTCTCAGAAATCTTCACCCTCTGACTCC |
| Ythdc2-F | GACGATGACGACAAGAAGCTTATGTCGAATGCATCTACAGCTTCA |
| Ythdc2-R | TGATGGATATCTGCAGAATTCTCAACTGGACTCCCACAGTGACA |
| Ythdc2-△HELICc-F | ACCTCATCCCAGGGATTTGCTTCCACCTCTTCA |
| Ythdc2-△HELICc-R | AAATCCCTGGGATGAGGTCCACGTCCACCCTCT |
| *hsa*-circYthdc2-F | CGGAATTCTAATACTTTCAGTGCTACACTGGAATTTGGAAAT |
| *hsa*-circYthdc2-R | CGGGATCCAGTTGTTCTTACCTGTAATGGCATTCTCAAAAGTTC |
| *hsa*-circYthdc2-FLAG-F | TACAAGGACGACGATGACAAGTGAAAGTTCTCTGGTTCAAACAAATG |
| *hsa*-circYthdc2-FLAG-R | GTCATCGTCGTCCTTGTAATCTCTAGATTTCCAAATTCCAGTGTAGC |
| *hsa*-circYthdc2-FLAG-m^6^A-1F | CTTGGCTTTGATCATGGATTACAAGGACGACG |
| *hsa*-circYthdc2-FLAG-m^6^A-1R | CCATGATCAAAGCCAAGTCTACTTTTTCATCATCGAA |
| *hsa*-circYthdc2-FLAG-ATG-1F | TGATCCCCGATTACAAGGACGACGATGACAAG |
| *hsa*-circYthdc2-FLAG-ATG-1R | CTTGTAATCGGGGATCAAATCCAAGTCTACTTTTTCATCA |
| *hsa*-circYthdc2-qRT-F | GGTAAGGTGAAAGAGA |
| *hsa*-circYthdc2-qRT-R | TAGCACTGTAATGGCA |
