# Supplementary figures and images for: CircYthdc2 generates polypeptides through two translation strategies to facilitate virus escape
Source: Cell Mol Life Sci. 2024 Feb 15;81(1):91. doi: 10.1007/s00018-024-05148-9 (PMC10869389; doi:10.1007/s00018-024-05148-9)

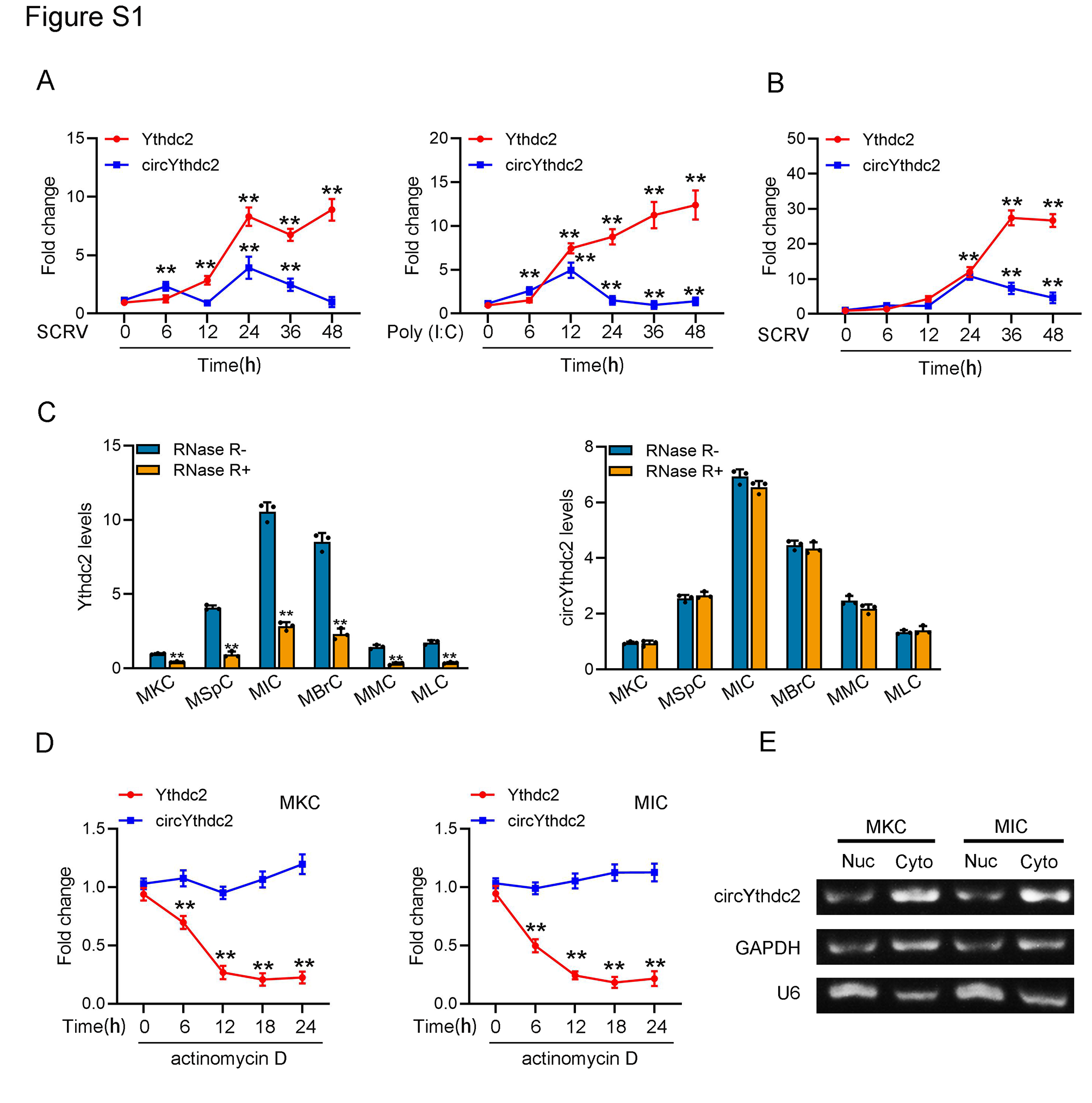

Supplement: Supplementary file 2 — Supplementary file2 (TIF 832 KB) [file 18_2024_5148_MOESM2_ESM.tif]
